# Malignant Transformation of Oral Leukoplakia and Proliferative Verrucous Leukoplakia and Its Biomarker Predictors: A Systematic Umbrella Review

**DOI:** 10.1002/hed.70073

**Published:** 2025-10-13

**Authors:** Mohammed Taib Fatih, Mohammed Khalid Mahmood, Balkees Taha Garib, Yad Mariwan Mohammed Amin, Ranjdar Mahmood Talabani, Tara Ali Rasheed, Handren Ameer Kurda, Balen Hamid Qadir, Zana Fuad Noori, Mohammed Aso Abdulghafor, Ariwan Othman Saeed, Herve Tassery, Delphine Tardivo, Romain Lan

**Affiliations:** ^1^ Department of Dentistry Komar University of Science and Technology Sulaymaniyah Iraq; ^2^ French National Center of Scientific Research (CNRS), French Blood Establishment (EFS), Bio‐Cultural Anthropology, Law, Ethics and Health Laboratory (ADES) Faculty of Medical and Paramedical Sciences, Aix‐Marseille University Marseille France; ^3^ College of Dentistry, Sulaymaniyah University Sulaymaniyah Iraq; ^4^ Department of Dentistry Tishk International University Sulaymaniyah Iraq; ^5^ Operative Dentistry and Endodontics, College of Dentistry University of Sulaimani Sulaymaniyah Iraq; ^6^ College of Dentistry, American University of Sulaymaniyah Iraq AUIS Sulaymaniyah Iraq; ^7^ Zhianawa Cancer Center Sulaymaniyah Iraq; ^8^ College of Medicine, Sulaymaniyah University Sulaymaniyah Iraq; ^9^ Dental School of Medicine, Conservative and Endodontic Department Aix‐Marseille University Marseille France; ^10^ Marseille Hospital APHM, IHU‐MEPHI Institute Marseille France

## Abstract

**Background:**

Oral leukoplakia (OL) represents the most common oral potentially malignant disorder globally, with highly variable reported malignant transformation (MT) rates creating challenges for evidence‐based clinical management.

**Objective:**

To systematically synthesize evidence on MT prevalence in OL and evaluate potential predictive biomarkers through an umbrella review of systematic reviews and meta‐analyses.

**Methods:**

Following PRISMA guidelines, we searched PubMed, MEDLINE, Scopus, and Embase databases through July 2025. Twenty‐seven systematic reviews encompassing more than 125,000 patients were included. Meta‐analyses were conducted using random‐effects models, with quality assessed using AMSTAR 2 and GRADE approaches.

**Results:**

Transformation rates were considerably higher in proliferative verrucous leukoplakia (48%) than in OL (6%). Females exhibited almost twice the MT rate of males (64% vs. 35%), while tongue lesions showed the highest site‐specific risk (39%). The most promising predictive biomarker with a moderate level of evidence quality was DNA aneuploidy.

**Conclusions:**

Because of its high malignant potential, OL necessitates risk‐based surveillance protocols. While the bulk of other predictors requires further investigation, DNA aneuploidy shows potential for clinical application.

## 1 Introduction

The World Health Organization defines oral leukoplakia (OL) as “a predominantly white plaque of questionable risk having excluded (other) known diseases or disorders that carry no increased risk for cancer” [1, 2, 3]. With prevalence rates that differ greatly among populations and geographical areas, it is the most prevalent oral potentially malignant disorder (OPMD) in the world [4, 5]. Epidemiological studies indicate that prevalence varies between 0.2% and 17% worldwide, with larger rates observed in developing countries and among people more exposed to traditional risk factors such as tobacco use, alcohol use, and betel quid chewing [6]. The condition mainly affects middle‐aged and older individuals, with a slight male predominance in most communities, although recent developments show changing demographic patterns [7]. OL is a significant disorder that requires standardized methods for diagnosis, risk assessment, and therapy due to regional variations in lifestyle factors, genetic predisposition, and exposure to carcinogenic agents [8, 9, 10].

The clinical significance of OL lies in its potential to develop into oral squamous cell cancer. As such, proper risk assessment is essential for patient care and surveillance procedures [11]. However, the reported MT rates are very different from one study to the next. For example, the MT rates for typical OL ranged from 3.5% [11] to 9.8% [12] and 9.8% [13], while proliferative verrucous leukoplakia (PVL) exhibits even more dramatic variation from 43.8% [14] to 65.8% [15]. In addition to the wide range of follow‐up periods from 6 months to 20 years and the time to malignancy from less than 1 year to more than 11 years [12, 13, 14, 15], the substantial heterogeneity in transformation rates makes it extremely difficult for clinicians to develop evidence‐based risk stratification and management strategies. Additionally, even though there have been several systematic reviews in this area, they have usually only looked at the prevalence of MT or possible predictive biomarkers, without offering a thorough synthesis of both.

This umbrella review systematically synthesizes evidence from all available systematic reviews (SRs) and meta‐analyses (MAs) to address critical knowledge gaps by providing pooled estimates of MT rates across various clinical variables and patient populations, while concurrently assessing the current state of evidence concerning potential predictive biomarkers. This study seeks to furnish clinicians and researchers with a comprehensive evidence‐based framework for comprehending MT risk in OL by integrating transformation prevalence data and predictor research. It aims to identify the most promising predictive tools and establish priorities for future research to enhance patient outcomes and clinical decision‐making.

## 2 Material and Methods

### 2.1 Protocol and Registration

This umbrella review was performed in accordance with the 2020 Preferred Reporting Items for Systematic Reviews and Meta‐Analyses (PRISMA) guidelines [16], and relevant methodological recommendations for umbrella reviews [17, 18]. The protocol for this review was prospectively registered in the International Prospective Register of Systematic Reviews (PROSPERO) with the registration number (CRD420251116225).

### 2.2 Eligibility Criteria

The research questions of this review were formulated according to the PECOS/T guideline as follows: (P) Problem: What is the pooled prevalence of MT in OL lesions? How does this prevalence change according to lesion subtype, lesion subsite, clinical appearance, sex, geography, cancer type, and dysplasia grade? How have the potential biomarkers been studied in SRs to predict the MT of OLs? (P) Population: The OL patients with or without MT. (E) Exposure: Presence or absence of MT and certain biomarkers. (C) Comparison: Intergroup group comparison with the controls or intragroup comparison between baseline and follow‐up. (O) Outcome: Prevalence of MT, under or over expression of biomarkers. (S) Study design: All SRs and MAs that are published in the English language and have presented their pooled analysis in proportion percentages or number of positive cases out of the studied sample. (T) Time: SRs and MAs that were published in PubMed, MEDLINE, Scopus, and Embase databases before July 2025.

There was no need for the definition of the variables like OL (and its subgroups), MT (and its subgroups), presence or absence of predictor biomarkers, because the included SRs have already defined these categories when selecting their primary studies for inclusion. Hence, we just collected each category of variables together.

Since there were only observational studies on these research questions, the included records did not contain any experimental studies. However, there were no restrictions on the observational study design within the included reviews, whether they were cross‐sectional, case–control, or cohort study designs.

All MAs that reported the number of MT cases out of the total number of OL patients were included. Those few MAs that presented their results in percentage, the point estimate was used to calculate the number of cases. Moreover, all MAs of pre‐clinical, animal, and in vitro studies were excluded.

Since we couldn't conduct a pooled analysis of the predictor‐related data due to a lack of comparability, we also included the SRs without MAs in the evidence synthesis of this outcome. Furthermore, the SRs on OPMDs were also included because these share the same common biomarker predictors as OL.

Only SRs and MAs published in peer‐reviewed journals in the English language were included. Reviews that did not apply systematic methods (narrative review, scoping review, etc.), those that reported their results using non‐comparable statistical tools and units, or those investigating one of the variables of OL or MT without studying the other were excluded. Conference abstracts, dissertations, editorials, or any other evidence not published in peer‐reviewed journals were excluded to ensure the credibility of the evidence identified.

### 2.3 Search Strategy

To find relevant MA and SRs evaluating the research question, the electronic databases of PubMed, MEDLINE, Scopus, and Embase were used to conduct the search to include all the records released before July 2025.

A search strategy was established based on a combination of controlled vocabulary (MeSH terms) and keywords related to OL (and its subtypes), MT (and its subtypes), and systematic review/meta‐analysis. The full list of search terms and combinations is presented in Table S1. There were no restrictions on publication dates; however, only English articles that met the inclusion criteria were included.

### 2.4 Study Selection

All the records obtained from databases were uploaded into reference management software, and duplicates were excluded. Study selection was conducted in two parts by two independent reviewers (M. M. and B. Q.). The two‐part study selection process began (Part 1) with screening of titles and abstracts for potentially relevant studies based on eligibility criteria, and then (Part 2) the full‐text articles of potentially eligible studies were screened in detail for eligibility. SRs and MAs that met all eligibility criteria were selected for inclusion. Disagreements during the screening process were resolved by discussion between the two reviewers. Where agreement was not reached, a third reviewer (M. A.) made the final decision.

### 2.5 Data Extraction

Data extraction was undertaken with a structured Microsoft Excel spreadsheet created for this review. Data was extracted independently by two reviewers (M. M. and B. Q.) to reduce the potential for errors and minimize variability. Discrepant data extraction was resolved through discussion, with a third reviewer (M. A.) consulted when consensus could not be reached.

To provide a structured collection of data by clinical variables/parameters, different worksheets within the Excel file were developed for overall prevalence, OL subtypes, MT subsites, clinical appearance, sex, geography, and dysplasia grades and type of the cancer in MT.

Study characteristics such as first author's surname, year of publication, number of primary studies included in the quantitative and qualitative analysis, type of study design of the primary studies, sample size, population characteristics, and main findings of the studies were extracted.

Since the main effect size of this review was prevalence and proportion, the number of MT positive cases together with the total number of OL cases was extracted for the pooled analysis. However, for those studies that only presented the prevalence in percentage and 95% CIs without the exact number of MT cases, the point estimate is extracted to estimate this number.

### 2.6 Quality Assessment

The AMSTAR 2 (A Measurement Tool to Assess Systematic Reviews) tool was used to assess the methodological quality of the included SRs [19]. This is a specialized tool for evaluating the methodological quality of SRs that examine studies of healthcare interventions that are either randomized or non‐randomized. The 16 items in the AMSTAR 2 tool evaluate the following domains: protocol registration, thorough literature search, evaluation of the risk of bias in individual studies, and appropriate meta‐analysis techniques. The AMSTAR 2 tool assigns a quality rating of high, moderate, poor, or critically low to each review. Special importance is given to the seven domains that AMSTAR 2 defines as “critical.” Two reviewers (M. M. and B. Q.) carried out each evaluation separately, and any disputes were settled by discussion or, if required, involvement from a third reviewer (M. A.).

### 2.7 Data Synthesis and Analysis

To ensure methodological consistency across included outcomes, we utilized the DerSimonian and Laird random‐effects model [20]. Since all the outcomes were in percentages, the number of cases and the total number of the sample were taken and reanalyzed.

Heterogeneity among studies was assessed using the *I* ^2^ statistical test. A *p*‐value of less than 0.10 was considered statistically significant for heterogeneity. The *I* ^2^ values were interpreted following the guidelines from the Cochrane Handbook for Systematic Reviews of Interventions: values between 0% and 40% were considered possibly unimportant; 30%–60% as indicative of moderate heterogeneity; 50%–90% as substantial; and 75%–100% as considerable heterogeneity [21].

In addition, the presence of publication bias was assessed through statistical methods. Egger's regression test was applied to detect small‐study effects, with a *p*‐value of less than 0.05 indicating potential bias [22]. These methods gave a structural way to check how strong and reliable the pooled estimates were. All statistical analyses in this umbrella review were conducted using Cochrane's RevMan tool accessed online [23].

For the qualitative analysis of the potential predictors, several factors were taken into account during the systematic evidence synthesis, such as key results, number of included studies in the SRs, heterogeneity of the findings, publication bias, and quality of the SRs.

### 2.8 Certainty of Evidence Assessment

The GRADE (Grading of Recommendations Assessment, Development and Evaluation) approach was used to assess the certainty of the evidence for each of the outcomes included in this umbrella review. GRADE allows for a systematic and transparent approach to assessing the quality of evidence across studies, including factors related to study design, methodological rigor, consistency of findings, directness of evidence, and imprecision (and risk of publication bias) [24].

Despite the data included in this review coming from SRs and MAs of observational studies, which the GRADE approach would typically categorize as “low” certainty at the beginning, we also applied the modifying factors that upgrade or downgrade the certainty in the evidence. For example, we downgraded evidence if there were outcomes with marked inconsistency in findings, serious risk of bias in the primary studies, and when effect estimates were imprecise (e.g., with large confidence intervals). Conversely, we considered upgrading the evidence if the effect was large, or we felt all credible residual confounding would produce a lesser effect.

## 3 Results

### 3.1 Study Selection

**FIGURE 1:**
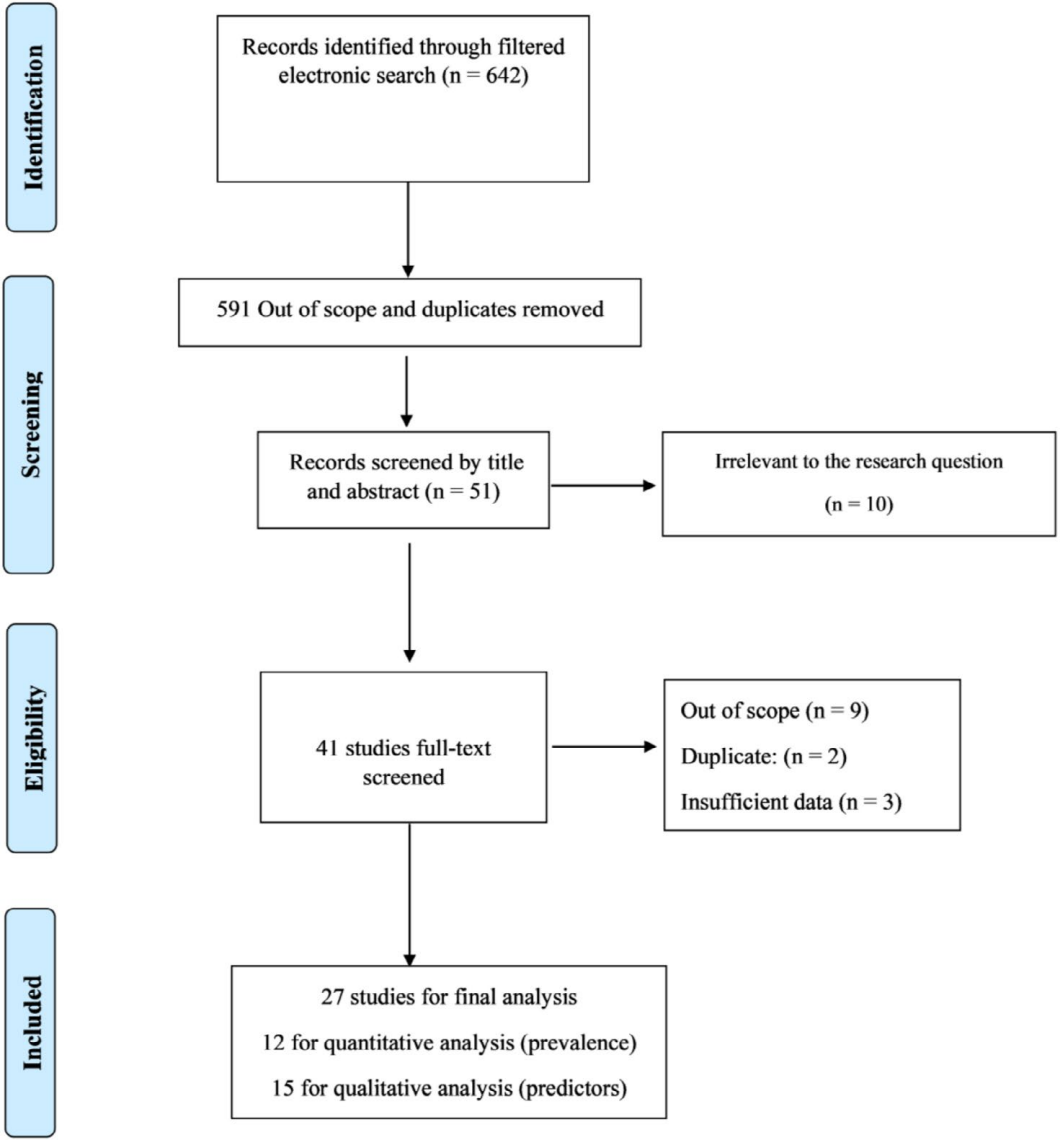
Study selection process. [Color figure can be viewed at wileyonlinelibrary.com]

Through a systematic search in PubMed, MEDLINE, Scopus, and Embase, a total of 642 records were detected. After deleting 591 out of scope and duplicates, 51 records remained for full‐text screening. Further, we excluded an additional 20 articles because the papers did not provide data relevant to the defined scope of the study (*n* = 15), were duplicates (*n* = 2), or had insufficient data (*n* = 3). Moreover, four reviews that were very close to being included were excluded [6, 25, 26, 27]. Table S2 presents these studies with their reasons for exclusion. Ultimately, 27 unique SRs met the inclusion criteria, of which 12 were included in the quantitative synthesis of the MT prevalence and 15 were included in the qualitative analysis of the potential predictors. The study selection process is shown in Figure 1.

### 3.2 Characteristics of the Included Systematic Reviews and Meta‐Analyses

Twelve studies [12, 13, 14, 15, 28, 29, 30, 31, 32, 33, 34, 35] encompassing 125 085 patients from a total of 383 were included. The average follow‐up time across studies ranged from 6 months to 20 years, with most studies reporting mean follow‐up periods between 5 and 8 years. Time to MT varied considerably, ranging from less than 1 year to over 11 years, with several studies reporting the mean transformation times of 3–4 years. The MT rates showed substantial variation by lesion type: OL demonstrated rates between 4.1% (homogeneous) and 22.9%, while PVL showed consistently higher transformation rates ranging from 45.8% to 65.8%. Table 1 shows the key characteristics of the included studies for MT prevalence.

**TABLE 1:** Key characteristics of the included studies for malignant transformation prevalence.

Studies	Number of studies	Total patients	Malignant cases	Follow‐up time	Time to malignancy	MT rate (95% CI)
Aguirre‐Urizar et al. (2021) [13]	24	16 604	1097	7.67 ± 5.02 years	3.2 ± 0.9 years (1.8–5.1)	OL: 9.8% (7.9–11.7)
Guan et al. (2023) [28]	26	6486	392	1–234 months	M: 3.58 ± 3.43 years, F: 3.95 ± 3.07 years	OL: 7.20% (5.40–9.10)
Iocca et al. (2020) [29]	36	17 830	748	0.5–20 years, 72/92 had a mean or median follow‐up greater than 2 years	MT rate per year: OL: 1.56% PVL: 9.3%	OL: 8.6% (5.1%–13%) PVL: 49.5% (26.7%–72.4%)
Ibanez et al. (2022) [15]	16	504	314	6.2 years (3.3–11.6)	4.16 years (< 1 to > 7)	PVL: 65.8% (55.3–76.2)
Mohideen et al. (2025) [30]	53	1159	561	6.28 ± 3.73 years	M: 3.58 ± 3.43 years, F: 3.95 ± 3.07 years	PVL: 48.4%
Paglioni et al. (2022) [31]	49	4767	477	6 months to 20 years	Not specified	Homogenous: 4.1% Non‐homogenous: 20%
Palaia et al. (2021) [32]	22	699	320	7.2 ± 6.3 years	4.1 years (mean)	PVL: 45.8%
Pimenta‐Barros et al. (2025) [33]	55	41 231	1575	Not specified	Not specified	OL: 6.64% (5.21–8.21)
Pinto et al. (2020) [34]	32	26 209	1762	5.5 years (1.5–20)	11–132 months	OL: 9.70% (7.80–11.70)
Ramos‐García (2021) [14]	17	474	211	2–14.5 years	6–108 months	43.87% (31.93–56.13)
Warnakulasuriya (2020) [4]	24	11 423	405	0.25–44 years	Not mentioned	3.5%
Vergier et al. (2025) [35]	14	58	27	3–18 years	3 years	GPVL: 47.75% (35.20–60.51)

Abbreviations: GPVL, gingival proliferative verrucous leukoplakia; MT, malignant transformation; OL, oral leukoplakia; PVL, proliferative verrucous leukoplakia.

Concerning the predictors, A total of 15 SRs [36, 37, 38, 39, 40, 41, 42, 43, 44, 45, 46, 47, 48, 49, 50] were included for the qualitative analysis of the potential biomarkers that may help in the prediction of MT of OPMDs. Four SRs investigated general biomarkers. The subject of three SRs was DNA aneuploidy. Two SRs were on MicroRNA. Moreover, gene expression, retinoblastoma protein (pRb), podoplanin, p53 and epidermal growth factor receptor (EGFR) were studied in one SR for each. Finally, one study compared the WHO and binary dysplasia grading systems to predict MT. Due to vast heterogeneity and lack of comparability of the studied exposures and outcomes we couldn't conduct a meta‐analysis. Key characteristics of these included studies and the systematic evidence synthesis are presented in Table S3.

### 3.3 Quality of the Systematic Reviews and Meta‐Analyses

Based on the AMSTAR 2 methodological quality assessment of 27 SRs examining MT of OL, the overall quality of included reviews was variable but generally acceptable. High‐quality reviews comprised 37% of the total (10/27). Moderate‐quality reviews accounted for 33% (9/27) and low‐quality reviews represented 30% (8/27). Critical methodological weaknesses were most commonly observed in domains related to risk of bias assessment (Q9), funding source reporting (Q10), publication bias investigation (Q15), and provision of excluded studies lists with justifications (Q7). Notably, all reviews adequately addressed research questions and inclusion criteria (Q1), study descriptions (Q8), heterogeneity discussions (Q14), and conflict of interest reporting (Q16), indicating strong performance in fundamental review components despite variability in advanced methodological rigor. Table S4 contains the full itemized evaluation of all the included studies.

### 3.4 Meta‐Analysis Results

In total, we conducted eight pooled analyses. Table 2 presents the meta‐analysis results.

**TABLE 2:** Meta‐analysis results of malignant transformation in oral leukoplakia patients.

Variable	No. of included estimates	Total no. of OL patients	Effect size (95% CI)	*p*	*I* ^2^, *p* heterogeneity	*p* Egger's test (publication bias)	Grade
MT prevalence by subtype	13	124 736				*p* = 0	Low
Leukoplakia	7	121 418	0.06 (0.04, 0.08)		99.4%, *p* < 0.0001		
PVL	6	3318	0.48 (0.42, 0.54)		90.2%, *p* < 0.0001		
Study quality							
Leukoplakia	7	121 418			99.4%, *p* < 0.0001		
Moderate	4	51 931	0.05 (0.04, 0.07)	0.49			
High	3	69 487	0.07 (0.03, 0.11)				
PVL	6	3318			90.2%, *p* < 0.0001		
Moderate	2	1123	0.44 (0.41, 0.48)	0.18			
High	4	2195	0.51 (0.42, 0.59)				
Subsite	30	15 159			99.1%, *p* = 0	0.60	Low
Tongue	5	2565	0.39 (0.22, 0.57)		98.8%, *p* < 0.0001		
Buccal mucosa	5	2565	0.21 (0.11, 0.33)		97.8%, *p* < 0.0001		
Floor of mouth	4	2276	0.06 (0.03, 0.11)		93.5%, *p* < 0.0001		
Gingiva	5	2453	0.23 (0.08, 0.43)		99%, *p* < 0.0001		
Lips	4	2276	0.04 (0.01, 0.08)		93%, *p* < 0.0001		
Palate	5	2565	0.05 (0.02, 0.09)		94.5%, *p* < 0.0001		
Multifocal	2	459	0.16 (0.00, 0.47)		98.1%, *p* < 0.0001		
Cancer type	8	3436		0.02	99.8%, *p* = 0	1	Low
SCC	4	1718	0.76 (0.44, 0.97)		99.2%, *p* < 0.0001		
VC	4	1718	0.24 (0.03, 0.56)		99.2%, *p* < 0.0001		
Sex	18	6472			97.5%, *p* < 0.0001		
Male	9	3236	0.35 (0.30, 0.40)	*p* < 0.0001	88.5%, *p* < 0.0001	0.9	Low
Female	9	3236	0.64 (0.59, 0.69)		87.2%, *p* < 0.0001		
OR (female vs. male)	4		1.35 (0.79, 2.32)	0.27	92.3%, *p* < 0.0001	0.88	
RR (female vs. male)	2		1.38 (1.19, 1.61)	*p* < 0.0001	0%, *p* = 0.80		
Geography	11	64 044		*p* = 0.006	99.5%, *p* = 0	0.045	Low
Europe	3	8941	0.17 (0.08, 0.28)		99.3%, *p* < 0.0001		
North America	3	8671	0.32 (0.06, 0.66)		99.8%, *p* < 0.0001		
Asia	3	45 831	0.04 (0.04, 0.05)		65.2%, *p* = 0.056		
Oceanica	2	601	0.07 (0.00, 0.20)		95.3%, *p* < 0.0001		
Clinical appearance	6	15 362		0.08	84%, *p* = 0.001	0.22	Low
Homogenous	3	6511	0.04 (0.03, 0.06)		69.7%, 0.01	1	
Non‐homogenous	3	8851	0.13 (0.04, 0.27)		99.3%, *p* < 0.0001		
Dysplasia	9	8106		0.40	96.9%, *p* < 0.0001	0.31	Low
Mild	3	4370	0.05 (0.01, 0.11)		97.7%, *p* < 0.0001		
Moderate	3	2575	0.10 (0.03, 0.20)		97.6%, *p* < 0.0001		
Severe	3	1161	0.11 (0.03, 0.23)		95.5%, *p* < 0.0001		

Abbreviations: CI, confidence interval; MT, malignant transformation; OL, oral leukoplakia; OR, odds ratio; PVL, proliferative verrucous leukoplakia; RR, risk ratio.

**FIGURE 2:**
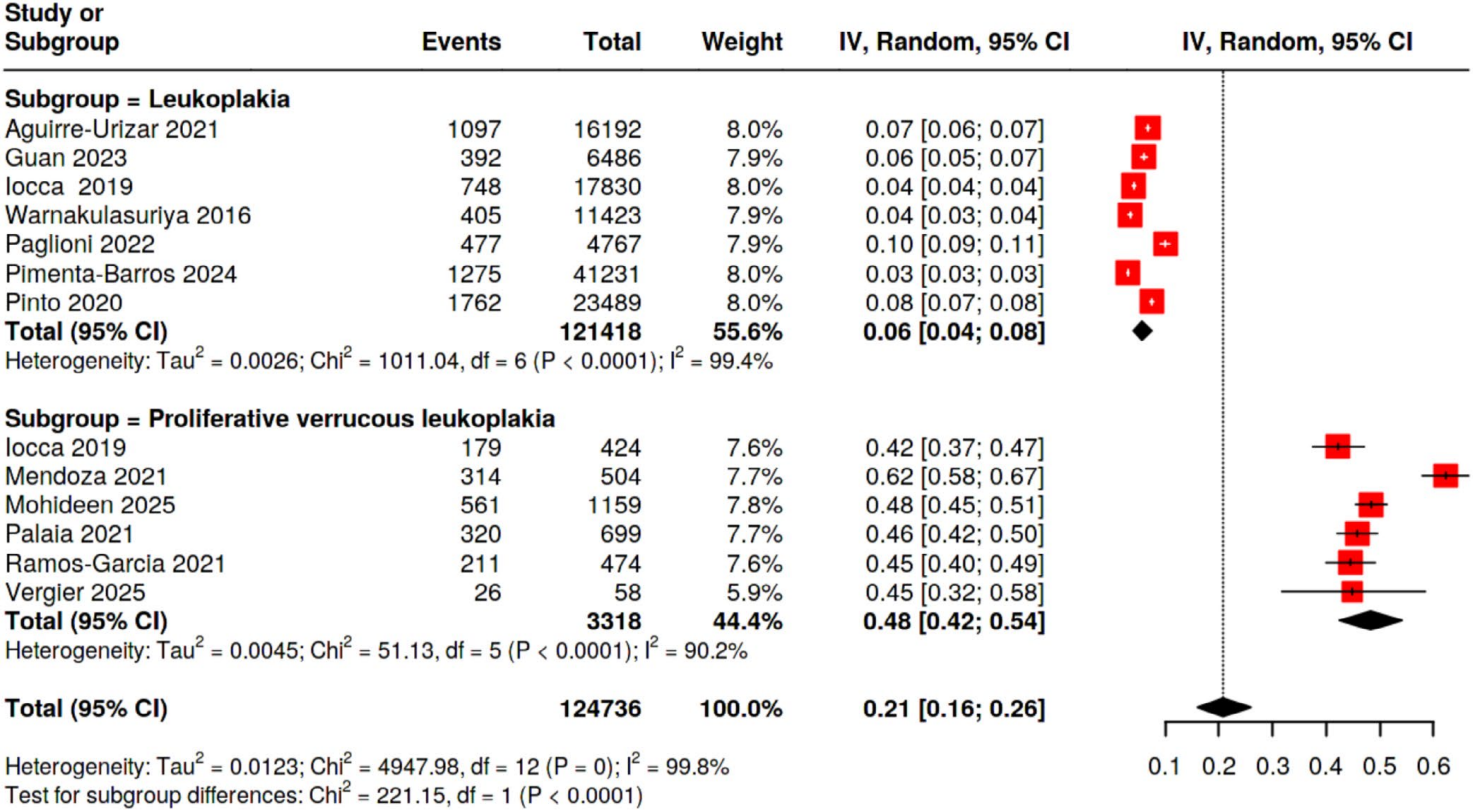
Forest plot of the prevalence of malignant transformation stratified by subtypes of leukoplakia and proliferative verrucous leukoplakia. [Color figure can be viewed at wileyonlinelibrary.com]

**FIGURE 3:**
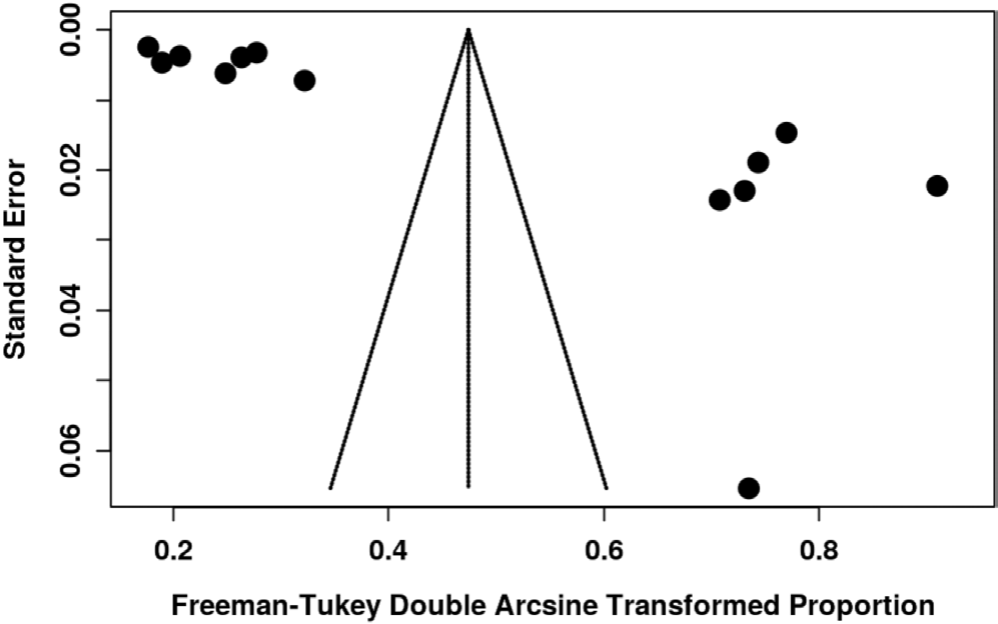
Funnel plot showing significant publication bias for the prevalence of malignant transformation among oral leukoplakia patients.

The subtype‐stratified analysis contained 13 estimates for OL and PVL. With significant heterogeneity (*I* ^2^ = 99.4%, *p* < 0.0001), the MT rate for OL was 0.06 (95% CI: 0.04, 0.08) (7 effect sizes, 121 418 patients). On the other hand, PVL demonstrated a significantly greater transformation rate of 0.48 (95% CI: 0.42, 0.54) including 3318 individuals and 6 estimations, with strong heterogeneity as well (*I* ^2^ = 90.2%, *p* < 0.0001) (Table 2 and Figures 2 and 3).

Regarding research quality, for OL, similar transformation rates of 0.05 (95% CI: 0.04, 0.07) and 0.07 (95% CI: 0.03, 0.11), respectively, were reported by moderate‐quality studies (4 estimates, 51 931 patients) and high‐quality studies (3 estimates, 69 487 patients) with a *p*‐value of 0.49 indicating no significant difference between the groups. The transformation rate for PVL was 0.44 (95% CI: 0.41, 0.48) for moderate‐quality studies (2 estimates, 1123 patients) and 0.51 (95% CI: 0.42, 0.59) for high‐quality studies (4 estimates, 2195 patients) with a *p* value of 0.18 indicating no significant difference between the groups (Table 2 and Figures S1 and S2).

Analysis of transformation rates across several oral subsites in 30 estimates involving 15 159 patients showed high heterogeneity (*I* ^2^ = 99.1%, *p* = 0). Across 5 estimations (2565 patients), the transformation rate was highest in the tongue (0.39, 95% CI: 0.22, 0.57), followed by the gingiva (0.23, 95% CI: 0.08, 0.43), and the buccal mucosa (0.21, 95% CI: 0.11, 0.33). The palate (0.05; 95% CI: 0.02, 0.09), lips (0.04; 95% CI: 0.01, 0.08), and floor of mouth (0.06; 95% CI: 0.03, 0.11) all showed lower rates. The transformation rate for multifocal lesions (2 estimations, 459 individuals) was 0.16 (95% CI: 0.00, 0.47) (Table 2 and Figure S3).

Eight estimates (3436 patients) for cancer types were considered, and the results indicated significant heterogeneity (*I* ^2^ = 99.8%, *p* = 0). Squamous cell carcinoma (SCC) accounted for 0.76 (95% CI: 0.44, 0.97) of the overall MT cases, and from the same number of estimates and patients, verrucous carcinoma (VC) exhibited a transformation rate of 0.24 (95% CI: 0.03, 0.56) (Table 2 and Figure S4).

**FIGURE 4:**
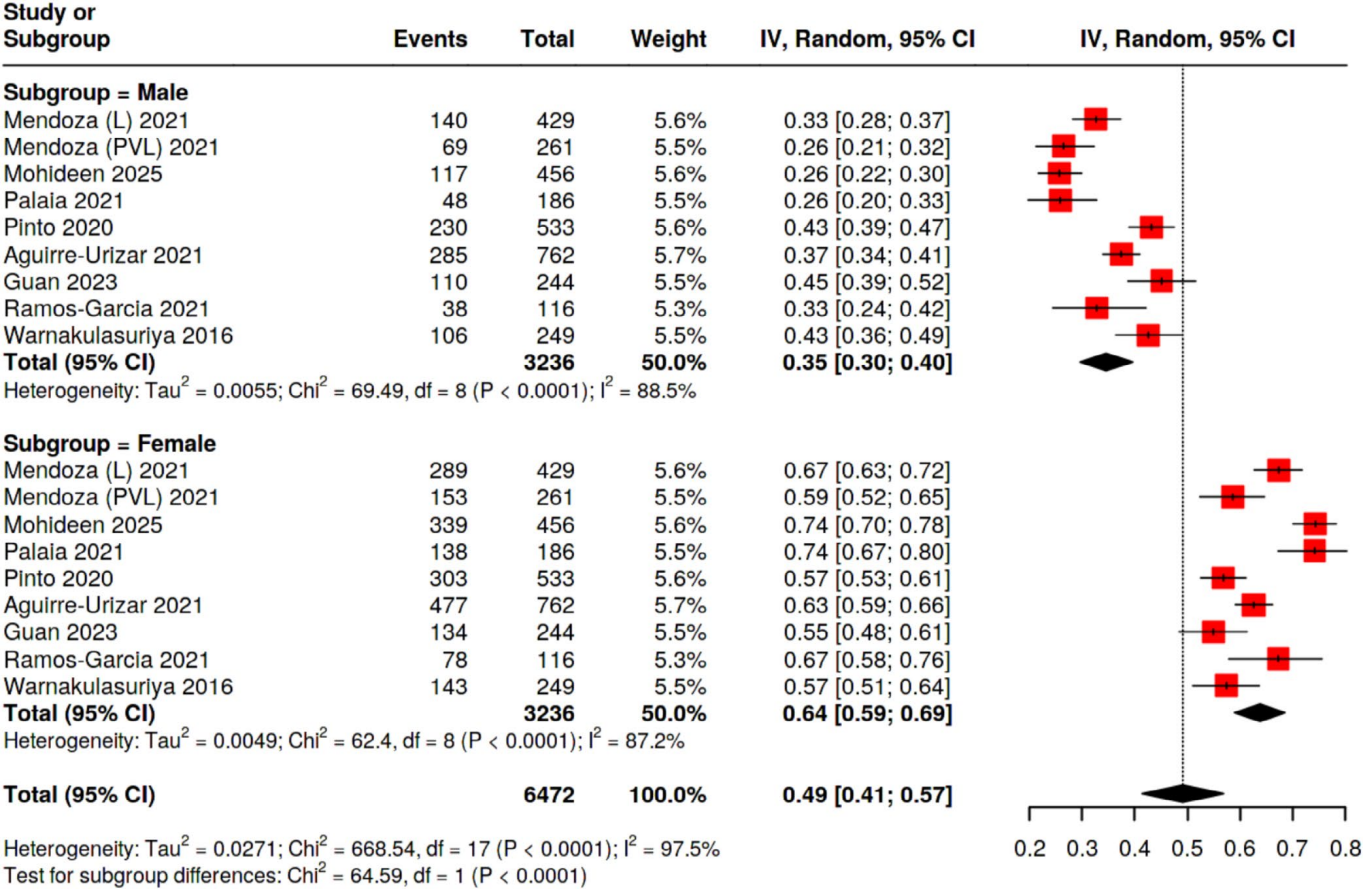
Forest plot of the malignant transformation rate in patients with oral leukoplakia stratified by sex. [Color figure can be viewed at wileyonlinelibrary.com]

Analysis by sex showed significant heterogeneity (*I* ^2^ = 97.5%, *p* < 0.0001) with 18 estimates (6472 patients). The transformation rate was 0.35 (95% CI: 0.30, 0.40) for males (9 estimates, 3236 patients) and 0.64 (95% CI: 0.59, 0.69) for females (9 estimates, 3236 patients). In comparison to males, females had an odds ratio (OR) of 1.35 (95% CI: 0.79, 2.32, *p* = 0.27) and a risk ratio (RR) of 1.38 (95% CI: 1.19, 1.61, *p* < 0.0001) for ML (Table 2 and Figure 4).

Significant heterogeneity was found in the geographic analysis, which included 11 estimations (64 044 patients) (*I* ^2^ = 99.5%, *p* = 0.006). North America had the highest transformation rate at 0.32 (95% CI: 0.06, 0.66), followed by Europe at 0.17 (95% CI: 0.08, 0.28) from 3 estimations (8941 patients). The rate was significantly lower in Oceanica (2 estimates, 601 patients) at 0.07 (95% CI: 0.00, 0.20) and Asia (3 estimates, 45 831 patients) at 0.04 (95% CI: 0.04, 0.05) (Table 2 and Figure S5).

Six estimations (15 362 patients) were included in the clinical appearance analysis, and there was a considerable amount of heterogeneity (*I* ^2^ = 84%, *p* = 0.001). According to three estimations (8851 patients), the transformation rate for non‐homogeneous lesions was 0.13 (95% CI: 0.04, 0.27) and that of homogeneous lesions was 0.04 (95% CI: 0.03, 0.06) from three estimations (6511 patients) (Table 2 and Figure S6).

Nine estimations involving 8106 patients were examined for dysplasia grade, and the results showed significant heterogeneity (*I* ^2^ = 96.9%, *p* < 0.0001). Mild dysplasia (3 estimates, 4370 patients) had a rate of 0.05 (95% CI: 0.01, 0.11), moderate dysplasia (3 estimates, 2575 patients) had a rate of 0.10 (95% CI: 0.03, 0.20), and severe dysplasia (3 estimates, 1161 patients) had the highest rate at 0.11 (95% CI: 0.03, 0.23) (Table 2 and Figure S7).

## 4 Discussion

To the best of our knowledge and as far as the literature search shows, this is the first umbrella review to collect and analyze all the previous SRs and MAs on the prevalence of MT in OL lesions and its predicting biomarkers aiming to shed light on the literature, snapshot the current situation of the field, detect the knowledge gaps, and pave the way for future studies to target interventions of higher quality.

### 4.1 Prevalence

The PVL showed a greater transformation rate of 48% (95% CI: 0.42–0.54), compared to the milder transformation rate of 6% (95% CI: 0.04–0.08) for conventional OL. This eight‐fold disparity highlights PVL's aggressiveness and bolsters its designation as a unique clinical entity in need of close observation and possibly more radical treatment. In the sensitivity analysis by study quality, studies with better quality resulted in a higher prevalence of MT compared with lower‐quality studies. Although the difference was not statistically significant, this could reveal the better ability of higher‐quality studies in the detection of MT lesions.

It should be noted that we only included studies of MT without known treatment and excluded the few studies on MT after treatment. For example, Dong et al. reported a prevalence of 4.5% after CO_2_ laser in 1864 OL patients [27]. Aguirre‐Urizar et al. documented 108 MT cases out of 947 OL cases treated with surgical excision, photodynamic therapy, occlusal adjustment, coaching tooth brushing method, medical therapies, and smoking cessation [13]. Comparative studies are needed to establish evidence on the effectiveness of treatment modalities on MT as an outcome.

The high rate of transformation seen in PVL is consistent with earlier research that described the disorder as a particularly aggressive kind of OL that is marked by high recurrence rates, resistance to treatment, and multifocal appearance [51, 52]. According to these results, people with PVL should get counseling regarding their markedly increased risk of developing cancer. They may also benefit from more regular monitoring schedules or preventive surgery [53, 54].

Significant variation in the rates of MT across several oral sites is revealed by our subsite analysis. The highest transformation rate was 39% (95% CI: 0.22–0.57) for the tongue, 23% (95% CI: 0.08–0.43) for the gingiva, and 21% (95% CI: 0.11–0.33) for the buccal mucosa. On the other hand, the transformation rates for lesions on the palate and lips were 5% and 4%, respectively. Our results recommend increased surveillance of leukoplakic lesions, especially on the tongue's posterior and lateral borders, which are known to be high‐risk regions for the development of oral SCC [55, 56]. Lip lesions are more frequently linked to sun exposure than to alcohol and tobacco use, which may explain the comparatively lower transformation rates seen in lip and palatal lesions [57].

A notable and statistically significant variation in the rates of MT between the sexes was found in the meta‐analysis. Females with a prevalence of 64% (95% CI: 0.59–0.69) had an MT rate nearly twice that of males, who had a rate of 35% (95% CI: 0.30–0.40). The result of the RR meta‐analysis (RR: 1.38, 95% CI: 1.19–1.61, *p* < 0.0001) with 38% higher risk for women supported the same MT dominance in females. Although the OR meta‐analysis from four estimates pointed in the same direction of evidence, the difference between the sexes was not significant (OR: 1.35, 95% CI: 0.79–2.32, *p* = 0.27). Despite this heterogeneity, the bulk of evidence supports higher rates of MT among female patients with OL. Further research is required to clarify whether this is a consequence of hormonal effects, physiological sensitivity, or shifts in the consumption of risk factors such as alcohol and smoking since this sex‐based disparity affects the follow‐up, management, and treatment of OL lesions.

The transformation rates varied greatly by geographical region, with Asia having the lowest rate at 4% (95% CI: 0.04–0.05), Europe coming in second at 17% (95% CI: 0.08–0.28), and North America at 32% (95% CI: 0.06–0.66). These gaps are most likely the result of differences in genetic vulnerability, risk factor exposures, diagnostic standards, and healthcare access practices, among other factors. The greater MT rates in European and North American populations compared to Asia could be attributed to differences in tobacco use patterns, hereditary factors, or early identification and intervention efforts [6, 58, 59]. However, given the likelihood of selection bias and regional variances in research methodology, these findings should be interpreted with caution.

Given that the MT rate for non‐homogeneous lesions was significantly higher (13%, 95% CI: 0.04–0.27) than for homogeneous lesions (4%, 95% CI: 0.03–0.06), our findings suggest that clinical appearance is an important prognostic factor. Current clinical guidelines [8, 11, 60] that stress the significance of lesion's form and appearance in risk assessment are supported by this study. Concerning the dysplasia grades, our research revealed that MT rates increased with greater grades of dysplasia (mild: 5%, moderate: 10%, and severe: 11%) in terms of histopathological grading, though these differences were not statistically significant (*p* = 0.40). The findings show a clear dose–response gradient, indicating that higher grades of dysplasia are associated with increased MT risk. Even though dysplasia grading is still a significant histopathological criterion [61, 62], this data raises the possibility that it may not be as predictive for MT as previously believed or that the grading schemes employed in various studies may not be sufficiently standardized [50]. Nevertheless, this conclusion is only based on the varying degrees of an existing dysplasia. Although we couldn't compare the presence and absence of dysplasia due to lack of data, it is highly likely that there will be a statistically significant difference between these categories in terms of MT.

With a rate of 76% (95% CI: 0.44–0.97) of transformed cases, SCC was the most common kind of malignancy, followed by VC at 24% (95% CI: 0.03–0.56). This distribution has consequences for treatment planning and prognosis evaluation, and it is consistent with the recognized spectrum of oral cancers resulting from OPMDs [63, 64].

### 4.2 Potential Predictors

In this paper, we also tended to assess the potential predictors of MT in OPMDs by combining data from available SRs. Although we couldn't perform a meta‐analysis in this regard, we followed a systematic evidence synthesis method by integrating components like the key findings, heterogeneity among the studies, potential publication bias, and methodological quality of the studies. Ultimately, the GRADE tool was utilized to evaluate the evidence certainty. Our findings show that different biomarker categories have different levels of clinical promise, but they all point to difficulties in integrating molecular predictors into standard clinical procedures.

The findings of several SRs that looked at a broad range of biomarkers were not very consistent. In their analysis of 32 included primary research, Huang et al. found that there were significant differences between OL patients and healthy controls in blood lipid‐bound sialic acid (LSA) and total sialic acid (TSA), as well as salivary interleukin (IL‐6) and tumor necrosis factor (TNF‐α) [36]. However, citing diverse research with small sample numbers and poor data reporting, Villa's evaluation of 25 studies revealed insufficient longitudinal evidence for validated predictive biomarkers [37]. Despite the large body of evidence, Celentano's thorough analysis of 54 research studies found that no one biomarker provided enough support for clinical risk categorization [40]. Although findings from several studies were inconsistent, podoplanin, DNA ploidy/chromosomal instability, stem cell markers, and p53 were the most intriguing possibilities found. The GRADE evidence quality for this category was rated as “very low” because of methodological flaws and publication bias issues.

Three SRs investigated the role of DNA aneuploidy in the MT prediction of OL lesions. Aliazari et al. in their meta‐analysis reported that lesions with aneuploidy had more than threefold higher risk for MT (RR = 3.12, 95% CI: 1.86–5.24), while OL lesions that exhibit diploid characteristics have 82% lower likelihood for their OL to progress toward malignancy [41]. Moreover, Annapoorani's SR that contained 30 primary studies documented that 93% of studies demonstrated a directly proportional relationship between DNA aneuploidy and MT [42]. The most convincing was Thakkar's meta‐analysis, which showed that aneuploid OL had a hazard ratio of 14.10 and a transformation rate of 75.2%, compared to only 8% for diploid lesions [43]. The biological justification is compelling since chromosomal instability is a fundamental characteristic of cancer [65, 66, 67]. Contrary to the other predictors, this category of DNA aneuploidy had lower publication bias and heterogeneity that led to a “moderate” quality of evidence as measured by the GRADE tool.

Kaunein et al.'s SR of 18 research studies reported nine continuously dysregulated miRNAs, especially miR‐21 and miR‐31 [44]. Performance metrics from Maheswaria's review, measured by area under the curve (AUC), revealed that miRNA‐184 (AUC = 0.86), miRNA‐21 (AUC = 0.73), and miRNA‐145 (AUC = 0.68) showed potential for early cancer detection [45]. However, this evidence gained a very low GRADE quality.

Normally, the most comprehensive biomarker approach is gene expression profiling, although there are some translation issues [68]. Among the included SRs, only one research studies gene expression of OL aiming for MT prediction. The synthesis of 15 included primary studies resulted in no specific genetic alterations that may help clinicians to differentiate between higher risk OL lesions from the lower risk ones [46]. The inherent technical complexity, resource requirements, and testing challenges of genome‐wide analysis hinder clinical deployment. Confidence in particular gene signatures is limited by the exploratory nature of the majority of studies and the absence of independent validation [69, 70]. Therefore, this outcome received a “very low” GRADE evidence quality.

A meta‐analysis of six studies (including 330 patients) by López‐Ansio et al. [71] revealed encouraging results regarding retinoblastoma protein (pRb) reduction, with moderate GRADE evidence quality. Loss of pRb expression was significantly associated with higher transformation risk (RR = 1.92, 95% CI: 1.25–2.94, *p* = 0.003), with effects being more noticeable in leukoplakia subgroups (RR = 2.00). The role of pRb in regulating the cell cycle makes the biological argument strong [72, 73]. Notably, this biomarker showed no heterogeneity (*I* ^2^ = 0%) and a low risk of publication bias, making it one of the more clinically ready markers discovered.

Podoplanin demonstrated a strong predictive value with moderate GRADE evidence quality (546 patients) in Monteiro's meta‐analysis of six studies. With no statistical heterogeneity (*I* ^2^ = 0%), the pooled hazard ratio for malignant development with high podoplanin expression was 3.72 (95% CI: 2.40–5.76, *p* < 0.00001) [38]. The scientific rationale is compelling given podoplanin's role in cell migration and the epithelial–mesenchymal transition [74, 75]. However, the limited size of individual studies and the lack of covariable data are two important drawbacks.

The meta‐analysis conducted by Ramos‐García on 24 trials showed that p53 overexpression had a moderate predictive value for 1210 patients. In leukoplakia subgroups, the approximately twofold elevated risk (RR = 1.88, 95% CI: 1.39–2.56, *p* < 0.001) persisted. However, clinical utility is limited by strong publication bias (Egger's test *p* = 0.01) and moderate heterogeneity (*I* ^2^ = 56%) [48]. Although the relationship seemed to be unaffected by the severity of dysplasia, implementation potential is impacted by methodological uniformity issues. Therefore, this outcome scored a “low” GRADE evidence quality. The protein p53, encoded by the TP53 gene, plays a crucial role as a tumor suppressor, acting like the “guardian of the genome”. Indeed, p53's underexpression is recorded in several steps of carcinogenesis [76, 77].

Cívico‐Ortega's meta‐analysis of 8 research studies (653 patients) revealed a strong correlation between transformation risk and EGFR overexpression [49]. The RR of 2.17 (95% CI: 1.73–2.73, *p* < 0.001) showed low risk of publication bias and no heterogeneity (*I* ^2^ = 0%). The biological rationale appears convincing given EGFR's role in invasion and proliferation pathways [78, 79]. However, standardizing immunohistochemical evaluation techniques is still difficult for clinical use. According to GRADE, this category had a moderate level of evidence quality.

Silva et al. compared the WHO and binary dysplasia grading systems. The binary and WHO systems predicted 31% and 40% MT rates, respectively, for severe dysplasia or carcinoma in situ lesions, and the difference between the two systems was not statistically significant (OR = 2.02; 95% CI: 0.88–4.64) [50]. This suggests that while grading dysplasia is an important histopathological feature, standardized grading procedures and improved inter‐observer agreement are necessary to boost its prognostic usefulness for MT [80].

### 4.3 Clinical Implications and Future Perspectives

Since PVL's MT rate (48%) differs eight times from conventional OL (6%), different approaches are needed. PVL requires close monitoring and timely expert referral. More site‐specific risk assessment is required for tongue lesions, which require immediate management due to their higher MT rate (39%) than palatal/lip lesions (4%–5%). Because female MT rates are greater (64% vs. 35% for males), sex‐specific surveillance measures are necessary. The threefold greater transformation rates (13% vs. 4%) in non‐homogeneous lesions highlight the necessity of a thorough clinical examination.

Research priorities include analyzing regional disparities (4% in Asia vs. 32% in North America) and developing standardized protocols that consider established risk factors, particularly alcohol and smoking. Combining clinical traits with molecular biomarkers and AI‐assisted analysis may enable personalized risk assessment. Large‐scale prospective studies are required to validate these findings and promote evidence‐based management.

The variations in GRADE evidence quality across different biomarker categories highlight the need for more rigorous study designs, standardized methodologies, and larger sample sizes to advance the field toward clinically applicable predictive tools for MT risk assessment.

### 4.4 Strength and Limitations

This umbrella review collected all the evidence from the available SRs and MAs on the MT of more than 125 000 OL patients with many clinical details like lesion subtype, lesion subsite, sex, geography, clinical appearance, and dysplasia grade as well as potential biomarkers that may be helpful in the prediction of MT. Besides, the most rigorous techniques of systematic evidence synthesis were employed to assess the current state of the literature.

However, this review had some limitations also. The conversion of a few studies that only documented percentage without exact numbers of OL and MT cases might have led to some imprecision in the effect sizes. A significant difference between original research is indicated by strong heterogeneity across meta‐analyses (*I* ^2^ = 84%–99.8%), which restricts the generalizability of pooled values. Some analyses may have overestimated transformation rates due to publication bias, which occurs when negative research is underrepresented. The methodological shortcomings in primary research, such as insufficient sample numbers and a lack of established methods, are reflected in the largely “low” GRADE evidence quality. Lastly, methodological heterogeneity prevented pooled analysis for the biomarker predictors.

## 5 Conclusion

With a prevalence of 48%, MT in patients with PVL is eight times higher than that of conventional OL (6%), requiring close monitoring of these patients. Anatomical site has a significant impact on transformation potential; lesions of the tongue (39%) have the highest rates compared to those of the palatal and lip regions (4%–5%). Women were almost twice as likely as men to undergo transformation (64% vs. 35%), and MT among non‐homogeneous lesions is three times more common than homogeneous ones (13% vs. 4%). Hence, OL can be defined as an established high‐risk precancerous condition.

DNA aneuploidy stands out as the most therapeutically promising biomarker among putative predictive biomarkers. It consistently produced positive results and has a strong scientific justification to be combined with traditional histological evaluations. Although podoplanin, retinoblastoma protein, and EGFR are examples of protein biomarkers with moderate evidence quality, their clinical application necessitates standardized screening procedures. However, the majority of biomarker categories displayed low evidence quality due to methodological limitations, small sample sizes, and publication bias. To validate these biomarkers and move closer to clinically useful predictive tools for MT risk assessment, large‐scale, carefully planned prospective studies with standardized methodologies are urgently needed.

## Ethics Statement

The authors have nothing to report.

## Consent

The authors have nothing to report.

## Conflicts of Interest

The authors declare no conflicts of interest.

## Supporting information


**Table S1:** The search strategy of the umbrella review
**Table S2:** List of excluded studies with reasons
**Table S3:** Included studies for potential predictors of malignant transformation of oral potentially malignant disorders
**Table S4:** Methodological quality assessment of the included systematic reviews using AMSTAR 2
**Figure S1:** Forest plot of the prevalence of malignant transformation in patients with oral leukoplakia according to study quality
**Figure S2:** Forest plot of the prevalence of malignant transformation in patients with proliferative verrucous leukoplakia according to study quality
**Figure S3:** Forest plot of the prevalence of malignant transformation in patients with oral leukoplakia stratified according to subsite
**Figure S4:** Forest plot of the transformed oral leukoplakia lesions stratified by cancer type
**Figure S5:** Forest plot of the prevalence of malignant transformation in patients with oral leukoplakia stratified by continents
**Figure S6:** Forest plot of the prevalence of malignant transformation in patients with oral leukoplakia stratified by clinical appearance
**Figure S7:** Forest plot of the prevalence of malignant transformation in patients with oral leukoplakia stratified by dysplasia grade

## Data Availability

The data that supports the findings of this study are available in the Supporting Information of this article.
