# Supplementary material for: Malignant Transformation of Oral Leukoplakia and Proliferative Verrucous Leukoplakia and Its Biomarker Predictors: A Systematic Umbrella Review
Source: Head Neck. 2025 Oct 13;48(1):246–60. doi: 10.1002/hed.70073 (PMC12703581; doi:10.1002/hed.70073)
Supplement: Supplementary file 1 — Table S1: The search strategy of the umbrella review Table S2: List of excluded studies with reasons Table S3: Included studies for potential predictors of malignant transformation of oral potentially malignant disorders Table S4: Methodological quality assessment of the included systematic reviews using AMSTAR 2 Figure S1: Forest plot of the prevalence of malignant transformation in patients with oral leukoplakia according to study quality Figure S2: Forest plot of the prevalence of malignant transformation in patients with proliferative verrucous leukoplakia according to study quality Figure S3: Forest plot of the prevalence of malignant transformation in patients with oral leukoplakia stratified according to subsite Figure S4: Forest plot of the transformed oral leukoplakia lesions stratified by cancer type Figure S5: Forest plot of the prevalence of malignant transformation in patients with oral leukoplakia stratified by continents Figure S6: Forest plot of the prevalence of malignant transformation in patients with oral leukoplakia stratified by clinical appearance Figure S7: Forest plot of the prevalence of malignant transformation in patients with oral leukoplakia stratified by dysplasia grade [file HED-48-246-s001.docx]

**Supplementary material for the article:**

**Malignant transformation of oral leukoplakia and its biomarker predictors: A systematic umbrella review**

| **Supplementary Table 1. The search strategy of the umbrella review** | | |
| --- | --- | --- |
| **Leukoplakia** | #1 ("Leukoplakia, Oral"[Mesh] OR "oral leukoplakia" OR "oral leucoplakia" OR "leukoplakia" OR "leucoplakia" OR "proliferative verrucous leukoplakia" OR "PVL" OR "verrucous leukoplakia" OR "oral potentially malignant disorder*" OR "oral potentially malignant lesion*" OR "OPMD*" OR "oral precancer*" OR "oral premalignant" OR "white patch*" OR "white plaque*" OR "oral white lesion*") |  |
| **Malignant transformation** | #2 ("Cell Transformation, Neoplastic"[Mesh] OR "Carcinogenesis"[Mesh] OR "malignant transformation" OR "malignant change" OR "malignant conversion" OR "cancer development" OR "cancer progression" OR "carcinogenesis" OR "neoplastic transformation" OR "progression to cancer" OR "transformation rate" OR "transformation risk" OR "oral cancer" OR "oral carcinoma" OR "squamous cell carcinoma" OR "SCC" OR "verrucous carcinoma" OR "oral squamous cell carcinoma" OR "OSCC") |  |
| **Biomarkers** | #3 ("Biomarkers, Tumor"[Mesh] OR "biomarker*" OR "predictor*" OR "predictive factor*" OR "prognostic factor*" OR "risk factor*" OR "DNA aneuploidy" OR "DNA ploidy" OR "chromosomal instability" OR "aneuploidy" OR "microRNA" OR "miRNA" OR "gene expression" OR "p53" OR "retinoblastoma protein" OR "pRb" OR "podoplanin" OR "EGFR" OR "epidermal growth factor receptor" OR "dysplasia" OR "dysplastic change*") |  |
| **Systematic review and meta-analysis** | #4 ("Systematic Review"[Publication Type] OR "Meta-Analysis"[Publication Type] OR "systematic review*" OR "meta-analysis" OR "meta analysis" OR "metaanalysis" OR "pooled analysis" OR "systematic literature review" OR "evidence synthesis" OR "umbrella review") |  |
| **Combination** | #5 #1 AND (#2 OR #3) AND #4 |  |

**Supplementary Table 2. List of excluded studies with reasons**

| **Studies** | **Reason** |
| --- | --- |
| Narayan (2016) [1] | Mixed the treated and non-treated malignant transformation cases. |
| González-Moles (2021) [2] | Focused on survival and mortality rather than prevalence and predictors. |
| Zhang (2023) [3] | Not studying the malignant transformation. |
| Dong (2019) [4] | Included only treated cases. |

**Supplementary Table 3. Included studies for potential predictors of malignant transformation of oral potentially malignant disorders**

| **Categories** | **No. of studies** | **Key results** | **Heterogeneity** | **Publication bias** | **Study quality by AMSTAR 2** | **GRADE** |
| --- | --- | --- | --- | --- | --- | --- |
| **General biomarkers** | 4 | Huang (2023) [5]:  32 studies included in quantitative analysis. Among the salivary biomarkers IL-6 and TNF-α exhibited statistically significant deviations in comparisons between HC versus OL and OL versus OC. Among the serum biomarkers, LSA and TSA exhibited statistically significant deviations in comparisons between HC versus OL and OL versus OC. | Significant for most of the outcomes | Not assessed due to small number of studies. | Moderate | Very low |
|  |  | Villa (2019) [6]:  25 studies included for qualitative analysis. This review identified insufficient longitudinal evidence to support validated prognostic biomarkers for OL. | Studies were heterogenous and tended to include small sample sizes, under-reported or variably reported main and confounding data. | Inclusion of subsets from chemoprevention trials may have introduced bias | Moderate |  |
|  |  | Monteiro (2025) [7]:  14 studies included in the qualitative analysis. Bmi-1,  Tipe-2, copy number alteration, DcR2, and Ki-67, PTHrP, podoplanin, and BubR1/Mad2 biomarkers presented independent significant value on MT. | Moderate | Not assessed or mentioned.  The findings may be influenced by selective reporting of positive results in the literature. | Moderate |  |
|  |  | Celentano (2020) [8]:  54 studies included for qualitative analysis. Despite wide evidence base and the large number of retrospective studies included, authors concluded that evidence was lacking to promote the advancement of any individual biomarker as an efficient tool for risk stratification of OL for clinical setting.  The most promising biomarkers across studies were podoplanin, DNA ploidy/chromosomal instability, stem cell markers (ALDH1, BMI-1, ABCG2), and p53. | Moderate heterogeneity:  Strongly positive (8 studies), moderately positive (6 studies), mixed results (11 studies), negative/neutral (4 studies). Remaining studies (25). | Not assessed and mentioned.  The findings may be influenced by selective reporting of positive results in the literature. | Moderate |  |
| **DNA aneuploidy** | 3 | Aliazari (2018) [9]:  5 studies included for quantitative analysis. Aneuploidy was found to be associated with a 3.12-fold increased risk to progress into cancer (RR=3.12, 95% CI 1.86-5.24). “No malignant progression” was more likely to occur in DNA diploid OPMD by 82% when compared to aneuploidy (RR=0.18, 95% CI 0.08-0.41). | Significant (RR=3.12, I^2^ = 65%, p = 0.02).  (RR=0.18, I^2^ = 91%, p < 0.00001). | Low risk | Low | Moderate |
|  |  | Annapoorani (2023) [10]:  30 studies included for qualitative analysis.  Positive: 28 out of 30 studies (93.3%)  Negative: 1 out of 30 studies (3.3%)  Neutral/conditional: 1 out of 30 studies (3.3%)  Use of DNA ploidy status can serve as an independent bio‑marker for predicting the malignant transformation of oral lesions. | Not significant | Not assessed | Low |  |
|  |  | Thakkar (2025) [11]:  12 studies included for quantitative analysis. OL with aneuploidy (75.2%) and diploid lesion (8%) had greater risk of MT. The HR of aneuploid OL had 14.10 times increased risk of MT compared with diploid OL. Detection of DNA aneuploidy status can help in predicting the MT of OL cases. | Mixed heterogeneity:  (RR=2.25, I^2^ = 95%, p < 0.00001).  (HR=14.10, I^2^ = 0%, p = 0.91). | Significant risk | High |  |
| **MicroRNA** | 2 | Kaunein (2021) [12]:  18 studies included for qualitative analysis. 9 miRNAs (especially MiR 21 and MiR 31) showed consistent dysregulation in all study when they were investigated. | Significant heterogeneity | Not assessed and mentioned.  The findings may be influenced by selective reporting of positive results in the literature. | Low | Very low |
|  |  | Maheswaria (2018) [13]:  6 studies included in the qualitative analysis. miRNA 184 upregulated with an area under the curve (AUC) of 0.86 and miRNA 21 with an AUC of 0.73 and downregulated miRNA 145 with an AUC of 0.68, which proved that these miRNAs are significant in detecting early malignancy in OPMD. | Moderate heterogeneity | Not assessed and mentioned.  The findings may be influenced by selective reporting of positive results in the literature. | Low |  |
| **Gene expression** | 1 | AbdulMajeed (2013) [14]:  15 studies included for qualitative analysis. Authors provided a list of common genes that may help focus selection of markers for the diagnosis and prognosis of OPMLs. | High heterogeneity | Not assessed and mentioned.  The findings may be influenced by selective reporting of positive results in the literature. | Low | Very low |
| **Retinoblastoma protein (pRb)** | 1 | López-Ansio (2025) [15]:  6 studies included for quantitative analysis recruiting 330 patients with OPMDs. The loss of pRb expression, was significantly associated with a higher malignant transformation risk of OPMDs (RR = 1.92, 95%CI = 1.25–2.94, p = 0.003). The leukoplakia subgroup retained this significant association (p = 0.006), being the OPMD where the loss of pRb expression showed the best predictive value for malignant transformation (RR = 2.00, 95%CI = 1.22–3.29). | OPMD: (6: studies: RR = 1.92, 95%CI: 1.25–2.94, I^2^ = 0, p = 0.58).  Leukoplakia: (2 studies: RR = 2.00, 95%CI:1.22–3.29, I^2^ = 0, p = 0.55). | Potentially no bias  No visual asymmetry in the funnel plot  Egger test p-value = 0.86. | High | Moderate |
| **Podoplanin** | 1 | Monteiro (2024) [16]:  6 studies included for quantitative analysis, that enrolled 546 patients  with OL, of whom 125 developed OC.  High expression of podoplanin carries a pooled HR of 3.72 (95% CI, 2.40–5.76; p < 0.00001) for MD. | No potential heterogeneity  (I^2^ = 0%, p = 0.53). | Not assessed and mentioned.  The findings may be influenced by selective reporting of positive results in the literature. | High | Moderate |
| **p53** | 1 | Ramos-García (2022) [17]:  24 studies (1,210 patients) met inclusion criteria. P53 overexpression was associated with a  statistically significant about 2 fold risk (RR = 1.88, 95 %CI = 1.39–2.56, p < 0.001). Leukoplakia maintained  this significant relationship after subgroup meta-analysis (p = 0.002). | Moderate heterogeneity:  I^2^ = 56%, p < 0.001 | Egger’s test p = 0.01.  Presence of small-study effects could not be potentially ruled out. | High | Low |
| **Epidermal Growth**  **Factor Receptor (EGFR)** | 1 | Cívico-Ortega (2025) [18]:  8 studies containing 653 patients with OPMD included.  EGFR upregulation was found to be significantly associated with an elevated malignant transformation risk of OPMD (RR = 2.17, 95%CI = 1.73–2.73, p < 0.001). | No potential heterogeneity:  I^2^ = 0%, p = 0.6 | No potential bias  Egger’s test p = 0.846. | High | Moderate |
| **Binary and WHO dysplasia grading systems** | 1 | Silva (2021) [19]:  4 studies included for qualitative and 3studies included for quantitative analysis.  WHO System: MT for severe dysplasia/carcinoma in situ = 40% (95% CI: 0.02-0.87)  Binary System: MT for high-risk lesions = 31% (95% CI: 0.00-0.84)  Overall comparison: No significant difference between systems (OR = 2.02; 95% CI: 0.88-4.64) | WHO system: I² = 92%  Binary system: I² = 97%  Overall comparison: I² = 0% (for OR analysis) | Not assessed | Moderate | Very low |

MT: malignant transformation, EFGR: epidermal growth factor receptor, OPMDs: oral potentially malignant disorders, OL oral leukoplakia, OC: oral cancer

**Supplementary Table 4. Methodological quality assessment of the included systematic reviews using AMSTAR 2**

| **Author (year) [reference]** | **Q1** | **Q2**  **(C)** | **Q3** | **Q4**  **(C)** | **Q5** | **Q6** | **Q7**  **(C)** | **Q8** | **Q9**  **(C)** | **Q10** | **Q11**  **(C)** | **Q12** | **Q13**  **(C)** | **Q14** | **Q15**  **(C)** | **Q16** | **Quality** |
| --- | --- | --- | --- | --- | --- | --- | --- | --- | --- | --- | --- | --- | --- | --- | --- | --- | --- |
| AbdulMajeed (2013) [14] | Y | Y | Y | PY | N | N | N | Y | N | N | NA | N | PY | Y | N | Y | Low |
| Aguirre-Urizar (2021) [20] | Y | Y | Y | Y | Y | Y | Y | Y | Y | N | Y | Y | Y | Y | N | Y | Moderate |
| Aliazari (2018) [9] | Y | N | Y | Y | Y | Y | N | Y | N | N | Y | N | PY | Y | Y | Y | Low |
| Annapoorani (2023) [8] | Y | Y | Y | PY | Y | Y | N | Y | Y | N | NA | N | PY | PY | N | Y | Low |
| Celentano (2021) [10] | Y | N | Y | Y | Y | Y | Y | Y | Y | N | NA | Y | Y | Y | N | Y | Moderate |
| Civico-Ortega (2025) [18] | Y | Y | Y | Y | Y | Y | Y | Y | Y | N | Y | Y | Y | Y | Y | Y | High |
| Guan (2023) [21] | Y | Y | Y | Y | Y | Y | N | Y | Y | N | Y | PY | PY | Y | Y | Y | Moderate |
| Huang (2023) [5] | Y | Y | Y | Y | Y | Y | N | Y | Y | N | Y | PY | Y | Y | N | Y | Moderate |
| Iocca (2020) [22] | Y | N | Y | Y | Y | Y | Y | Y | Y | N | Y | Y | Y | Y | N | Y | Moderate |
| Kaunein (2021) [12] | Y | N | Y | Y | Y | Y | Y | Y | N | N | NA | N | N | Y | N | Y | Low |
| Lopez-Ansio (2025) [15] | Y | Y | Y | Y | Y | Y | Y | Y | Y | N | Y | Y | Y | Y | Y | Y | High |
| Maheswaria (2018) [13] | Y | N | PY | PY | Y | Y | N | Y | Y | N | NA | PY | PY | N | N | Y | Low |
| Thakkar (2025) [11] | Y | Y | Y | Y | Y | Y | N | Y | Y | N | Y | PY | Y | Y | Y | Y | High |
| Mendoza (2022) [23] | Y | Y | Y | Y | Y | Y | N | Y | Y | N | Y | Y | Y | Y | Y | Y | High |
| Mohideen (2025) [24] | Y | Y | Y | Y | Y | Y | N | Y | Y | N | Y | PY | Y | Y | Y | Y | High |
| Monteiro (2024) [16] | Y | Y | Y | Y | Y | Y | Y | Y | Y | N | Y | Y | Y | Y | N | Y | High |
| Monteiro (2025) [7] | Y | Y | Y | Y | Y | Y | Y | Y | Y | N | NA | PY | Y | Y | N | Y | Moderate |
| Narayan (2016) [1] | Y | N | Y | PY | N | N | N | Y | N | N | PY | N | N | PY | N | Y | Low |
| Paglioni (2022) [25] | Y | Y | Y | Y | Y | Y | Y | Y | Y | N | Y | Y | Y | Y | N | Y | High |
| Palaia (2021) [26] | Y | Y | Y | Y | Y | Y | N | Y | Y | N | Y | Y | Y | PY | N | Y | Moderate |
| Pimenta-Barros (2025) [27] | Y | Y | Y | Y | Y | Y | Y | Y | Y | N | Y | Y | Y | Y | Y | Y | High |
| Pinto (2020) [28] | Y | Y | Y | Y | Y | Y | Y | Y | Y | N | Y | Y | Y | Y | Y | N | High |
| Ramos-Garcia (2021) [29] | Y | Y | Y | Y | Y | Y | Y | Y | Y | N | Y | Y | Y | Y | Y | Y | High |
| Ramos-Garcia (2022) [30] | Y | Y | Y | Y | Y | Y | Y | Y | Y | N | Y | Y | Y | Y | Y | Y | High |
| Silva (2021) [19] | Y | Y | Y | Y | Y | Y | Y | Y | Y | N | Y | PY | PY | Y | N | Y | Moderate |
| Warnakulasuriya (2020) [31] | Y | N | Y | Y | N | N | Y | Y | N | N | NA | NA | PY | Y | NA | N | Moderate |
| Vergier (2025) [32] | Y | Y | Y | Y | Y | Y | N | Y | Y | N | Y | Y | Y | Y | PY | Y | High |
| Villa (2019) [6] | Y | Y | Y | Y | Y | Y | N | Y | Y | N | NA | PY | Y | Y | N | Y | Moderate |
| **Ref: references, C: critical domain, N: No, NA: not applicable, PY: partially yes, Y: Yes. Q1: Did the research questions and inclusion criteria for the review include the components of PICO?, Q2: Did the report of the review contain an explicit statement that the review methods were established prior to the conduct of the review and did the report justify any significant deviations from the protocol?; Q3, Did the review authors explain their selection of the study designs for inclusion in the review?; Q4, Did the review authors use a comprehensive literature search strategy?; Q5 Did the review authors perform study selection in duplicate?; Q6, Did the review authors perform data extraction in duplicate?; Q7, Did the review authors provide a list of excluded studies and justify the exclusions?; Q8, Did the review authors describe the included studies in adequate detail?; Q9, Did the review authors use a satisfactory technique for assessing the risk of bias?; Q10, Did the review authors report on the sources of funding?; Q11, Did the review authors use appropriate methods for statistical combination of results?; Q12, Did the review authors assess the potential impact of RoB in individual studies on the results?; Q13, Did the review authors** **account for RoB in individual studies when interpreting/ discussing the results of the review?; Q14, Did the review authors provide a satisfactory explanation for, and discussion of, any heterogeneity?; Q15, Did the review authors carry out an adequate investigation of publication bias?; Q16, Did the review authors report any potential sources of conflict of interest?** | | | | | | | | | | | | | | | | | |


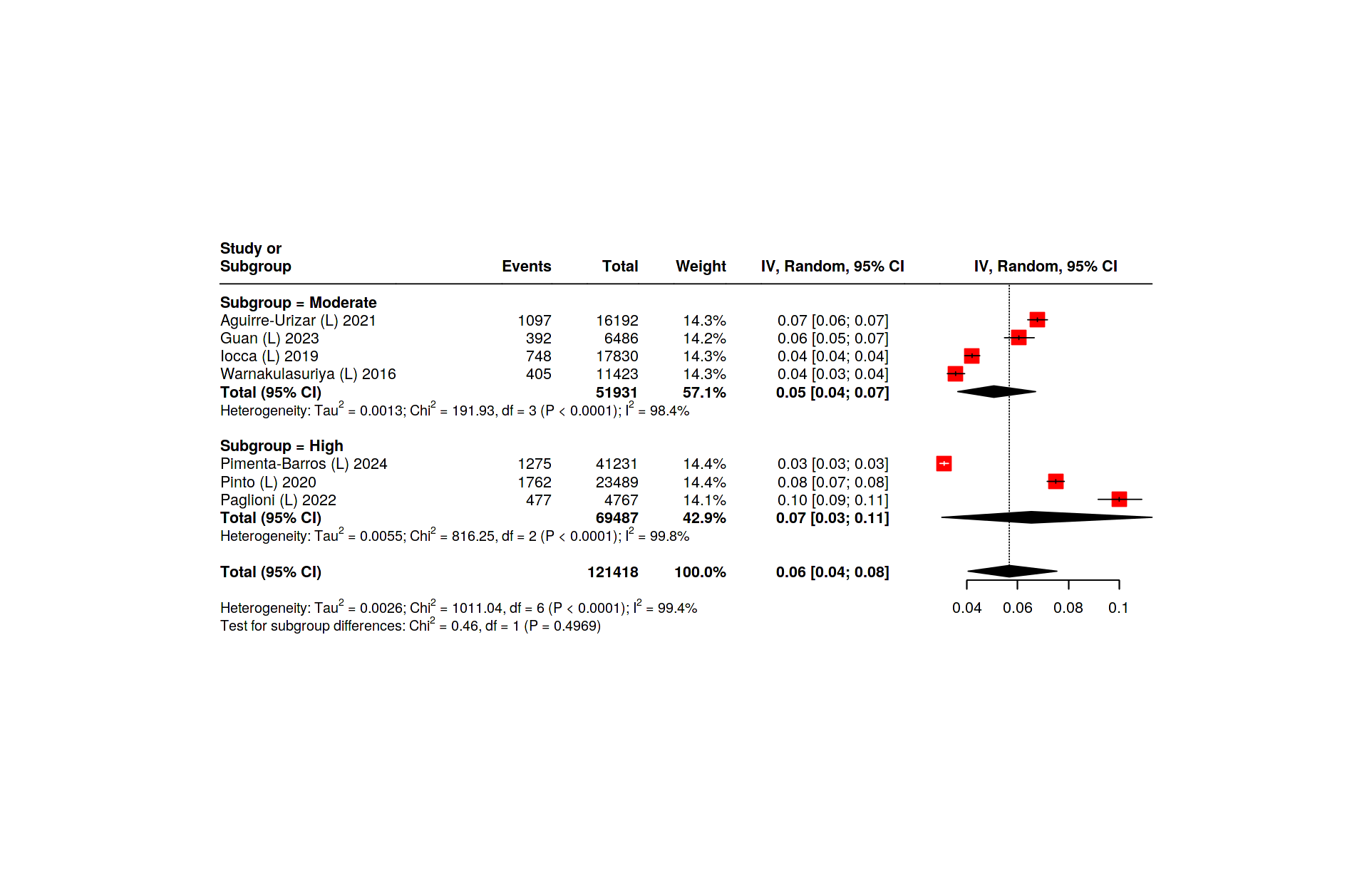


Supplementary Figure 1. Forest plot of the prevalence of malignant transformation in patients with oral leukoplakia according to study quality


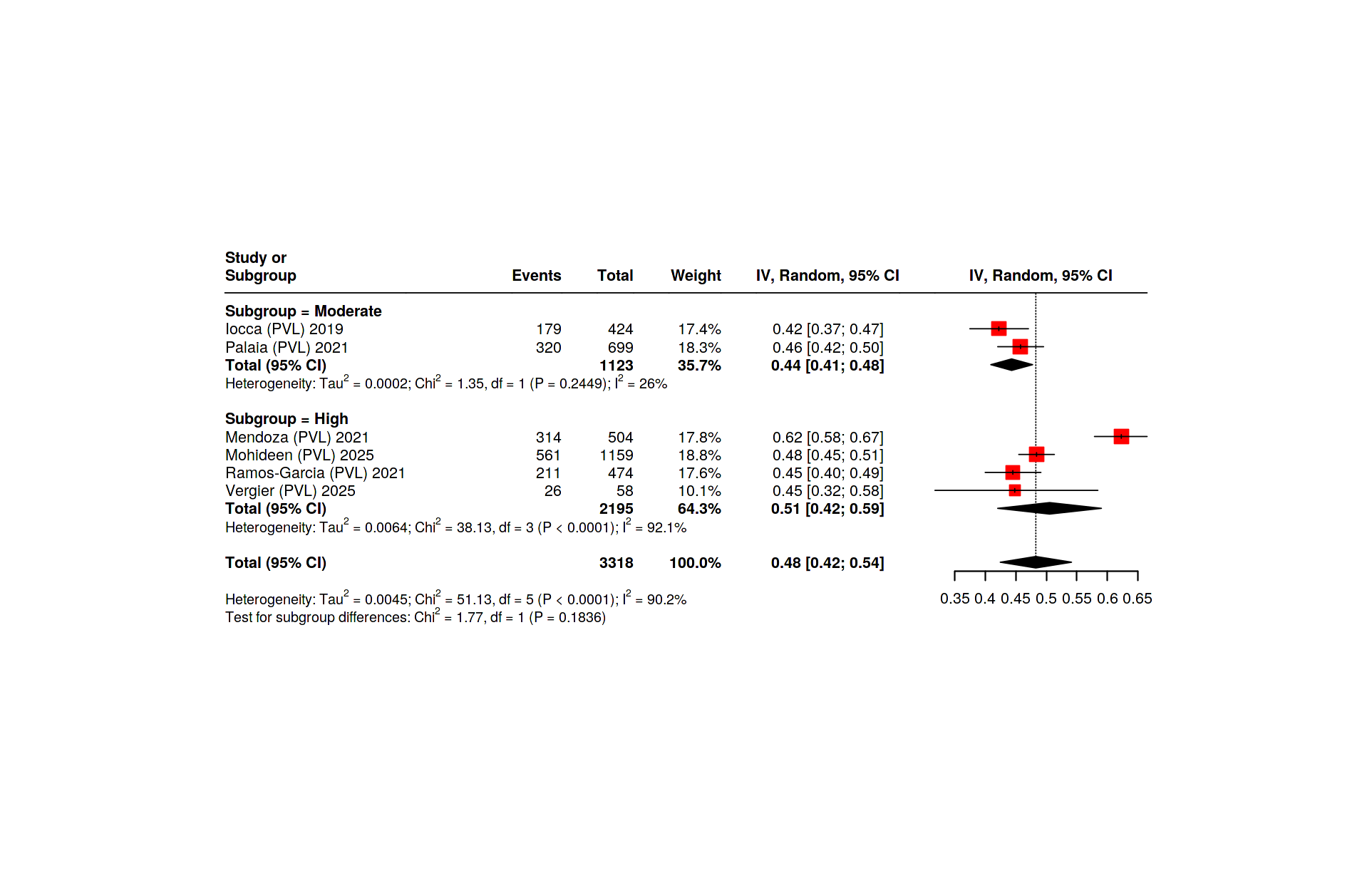


Supplementary Figure 2. Forest plot of the prevalence of malignant transformation in patients with proliferative verrucous leukoplakia according to study quality


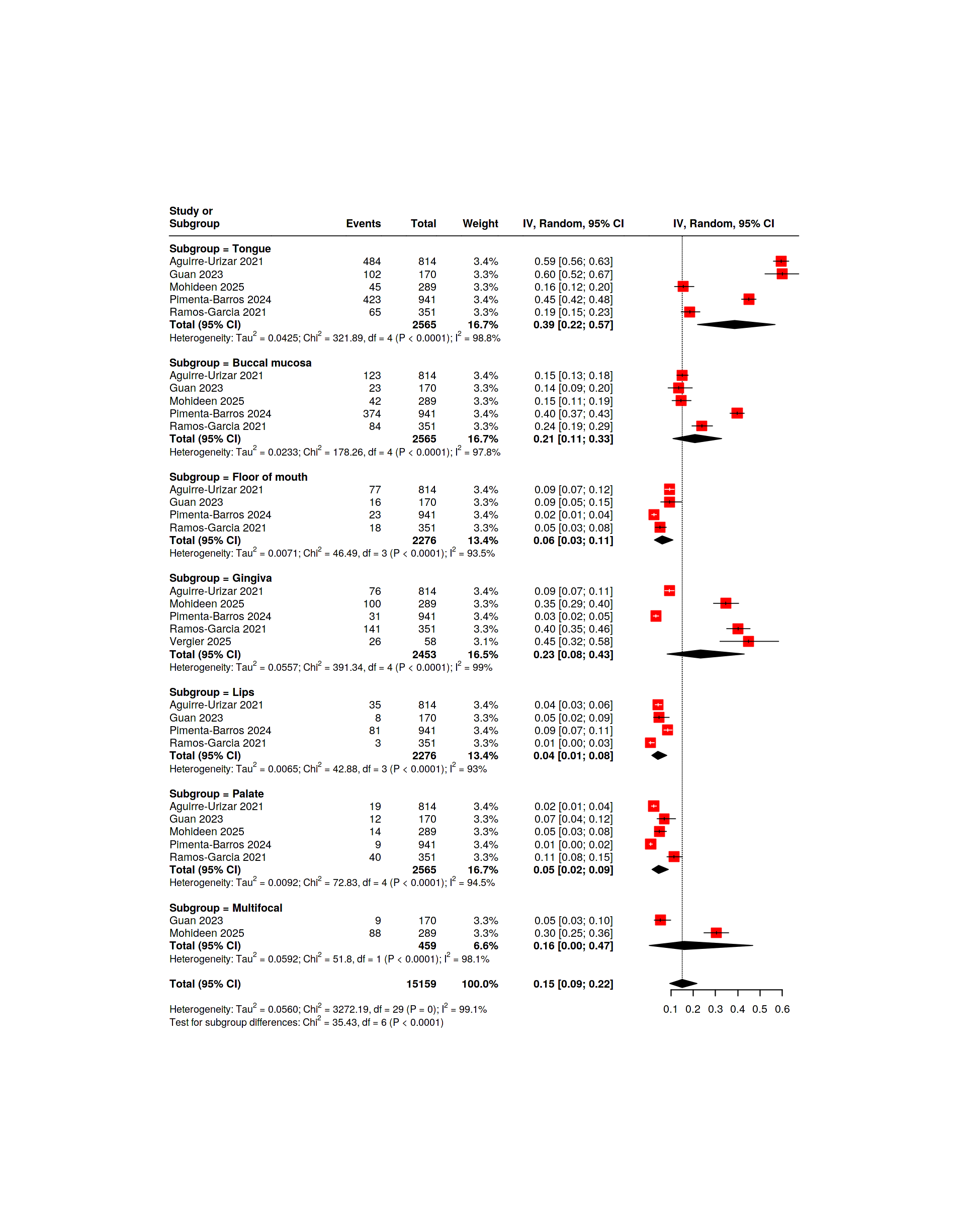


Supplementary Figure 3. Forest plot of the prevalence of malignant transformation in patients with oral leukoplakia stratified according to subsite


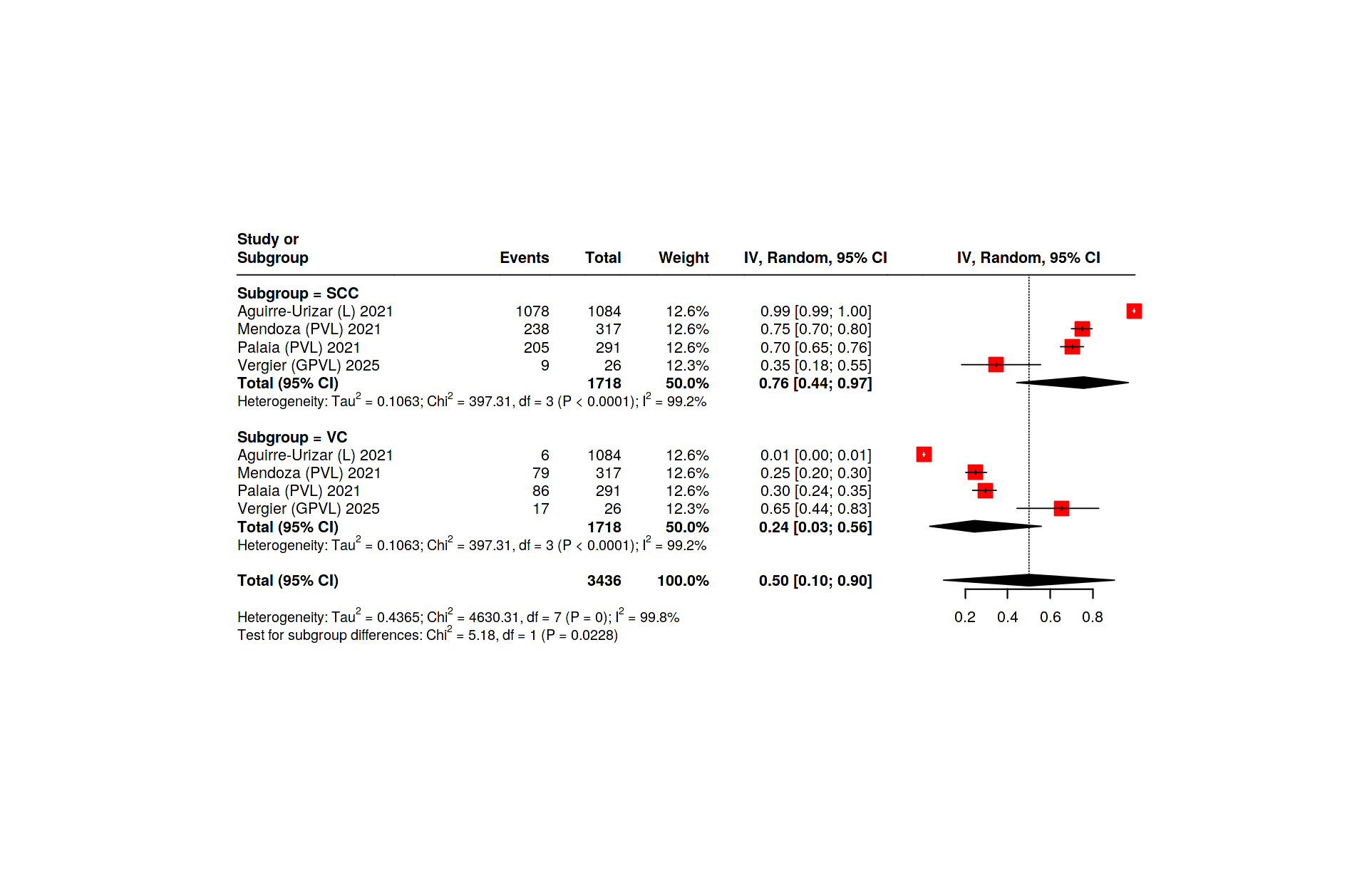


Supplementary Figure 4. Forest plot of the transformed oral leukoplakia lesions stratified by cancer type.

SCC: squamous cell carcinoma, VC: verrucous carcinoma


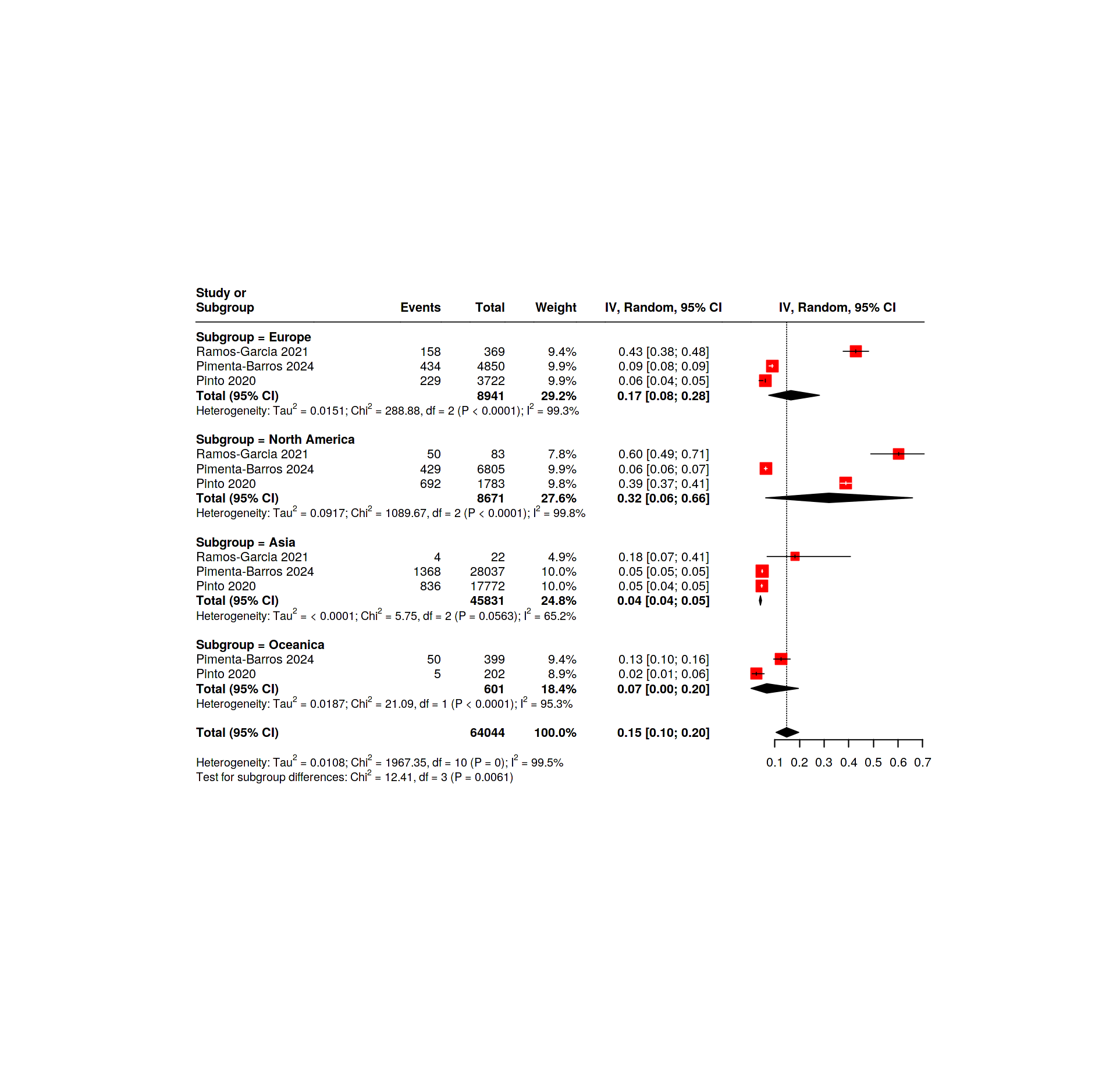


Supplementary Figure 5. Forest plot of the prevalence of malignant transformation in patients with oral leukoplakia stratified by continents.


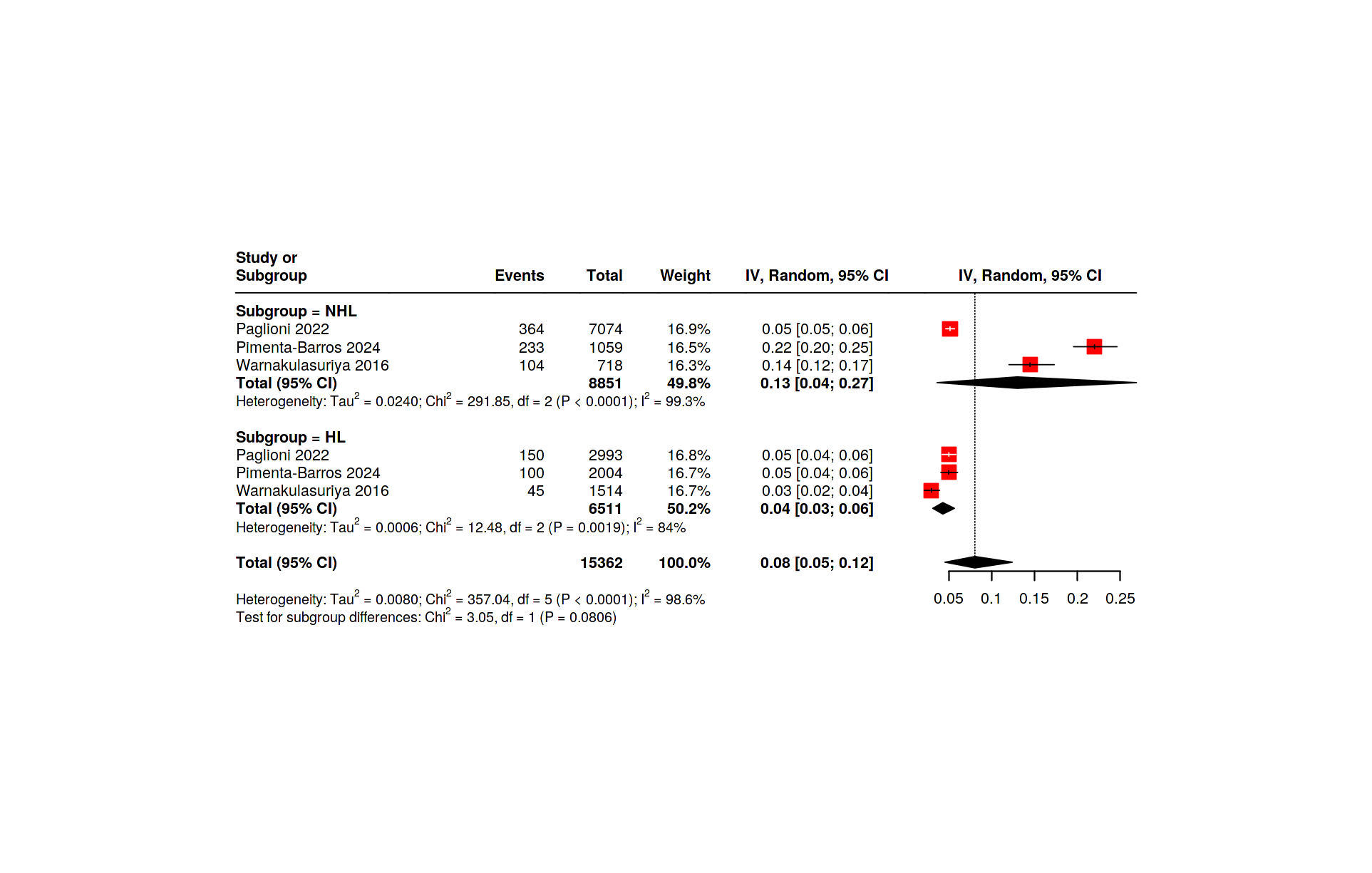


Supplementary Figure 6. Forest plot of the prevalence of malignant transformation in patients with oral leukoplakia stratified by clinical appearance.

HL: homogenous leukoplakia, non-homogenous leukoplakia


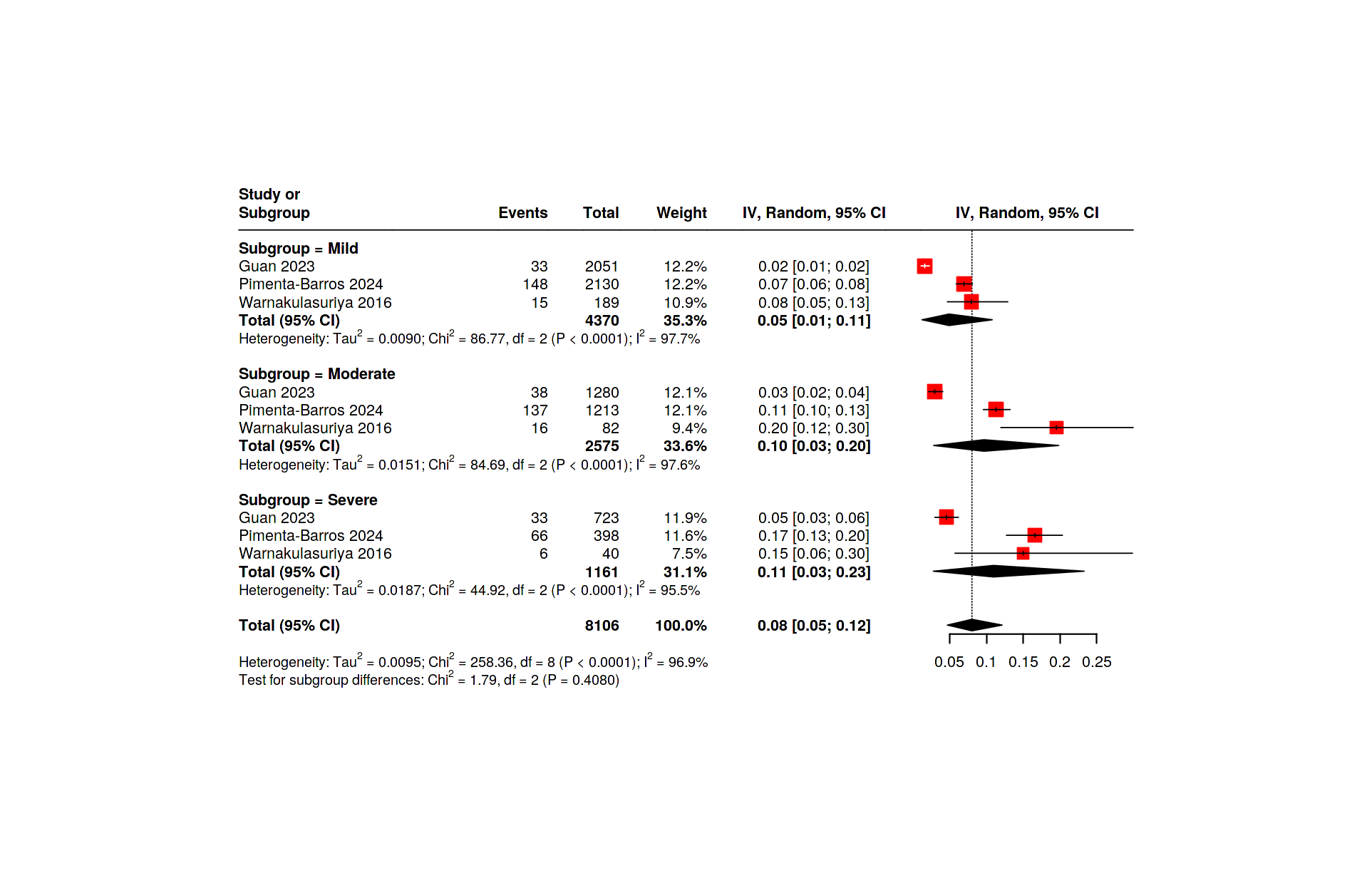


Supplementary Figure 7. Forest plot of the prevalence of malignant transformation in patients with oral leukoplakia stratified by dysplasia grade.

References:

1. Narayan T, Shilpashree S. Meta-analysis on clinicopathologic risk factors of leukoplakias undergoing malignant transformation. J Oral Maxillofac Pathol. 2016;20(3):354–61.

2. Gonzalez-Moles MA, Warnakulasuriya S, Ramos-Garcia P. Prognosis parameters of oral carcinomas developed in proliferative verrucous leukoplakia: a systematic review and meta-analysis. Cancers. 2021;13(19):4843.

3. Zhang C, Li B, Zeng X, Hu X, Hua H. The global prevalence of oral leukoplakia: a systematic review and meta-analysis from 1996 to 2022. BMC Oral Health. 2023;23(1):645.

4. Dong Y, Chen Y, Tao Y, Hao Y, Jiang L, Dan H, et al. Malignant transformation of oral leukoplakia treated with carbon dioxide laser: a meta-analysis. Lasers Med Sci. 2019;34(1):209–21.

5. Huang Y, Zhang Q, Guo Z, Deng G, Chen R, Zheng Y. Potential noninvasive biomarkers for the malignant transformation of oral leukoplakia: A systematic review and meta‐analysis. Cancer Med. 2023;12(13):14718–30.

6. Villa A, Celentano A, Glurich I, Borgnakke WS, Jensen SB, Peterson DE, et al. World Workshop on Oral Medicine VII: Prognostic biomarkers in oral leukoplakia: A systematic review of longitudinal studies. Oral Dis. 2019;25:64–78.

7. Monteiro L, Rocha E, Ferreira S, Salazar F, Pacheco JJ, Warnakulasuriya S. Tissue Biomarkers for Predicting the Risk of Oral Cancer in Patients Diagnosed With Oral Leukoplakia: A Systematic Review of the Past 4 Years. J Oral Pathol Med. 2025;54(5):283–9.

8. Celentano A, Glurich I, Borgnakke WS, Farah CS. World Workshop on Oral Medicine VII: Prognostic biomarkers in oral leukoplakia and proliferative verrucous leukoplakia—A systematic review of retrospective studies. Oral Dis. 2021;27(4):848–80.

9. Alaizari NA, Sperandio M, Odell EW, Peruzzo D, Al‐Maweri SA. Meta‐analysis of the predictive value of DNA aneuploidy in malignant transformation of oral potentially malignant disorders. J Oral Pathol Med. 2018;47(2):97–103.

10. Annapoorani S, Gururaj N, Balambigai VA, Prakash N, Hasinidevi P, Janani V. Assessment of ploidy status in oral potentially malignant disorders–a systematic review. J Pharm Bioallied Sci. 2023;15(Suppl 1):S86–92.

11. Thakkar N, Mane DR, Angadi PV. DNA Ploidy Status as a Predictor for Malignant Transformation in Oral Leukoplakia: A Systematic Review and Meta-Analysis. Oral Surg Oral Med Oral Pathol Oral Radiol. 2025;

12. Kaunein N, Ramani RS, Koo K, Moore C, Celentano A, McCullough M, et al. A systematic review of microRNA signatures associated with the progression of leukoplakia with and without epithelial dysplasia. Biomolecules. 2021;11(12):1879.

13. Maheswari TU, Venugopal A, Sureshbabu NM, Ramani P. Salivary micro RNA as a potential biomarker in oral potentially malignant disorders: A systematic review. Tzu Chi Med J. 2018;30(2):55–60.

14. AbdulMajeed AA, Farah CS. Gene expression profiling for the purposes of biomarker discovery in oral potentially malignant lesions: a systematic review. Clin Med Insights Oncol. 2013;7:CMO-S12950.

15. López-Ansio M, Ramos-García P, González-Moles MÁ. Predictive Value of the Loss of pRb Expression in the Malignant Transformation Risk of Oral Potentially Malignant Disorders: A Systematic Review and Meta-Analysis. Cancers. 2025;17(2):329.

16. Monteiro L, Mariano LC, Warnakulasuriya S. Podoplanin could be a predictive biomarker of the risk of patients with oral leukoplakia to develop oral cancer: A systematic review and meta‐analysis. Oral Dis. 2024;30(2):207–15.

17. Ramos-Garcia P, Gonzalez-Moles MA, Warnakulasuriya S. Significance of p53 overexpression in the prediction of the malignant transformation risk of oral potentially malignant disorders: A systematic review and meta-analysis. Oral Oncol. 2022;126:105734.

18. Cívico-Ortega JL, Ramos-García P, González-Moles MÁ. Significance of Epidermal Growth Factor Receptor (EGFR) upregulation in the prediction of the malignant transformation risk in oral potentially malignant disorders: a systematic review and meta-analysis. Front Oral Health. 2025;6:1578561.

19. de Freitas Silva BS, Batista DCR, de Souza Roriz CF, Silva LR, Normando AGC, dos Santos Silva AR, et al. Binary and WHO dysplasia grading systems for the prediction of malignant transformation of oral leukoplakia and erythroplakia: a systematic review and meta-analysis. Clin Oral Investig. 2021;25(7):4329–40.

20. Aguirre‐Urizar JM, Lafuente‐Ibáñez de Mendoza I, Warnakulasuriya S. Malignant transformation of oral leukoplakia: Systematic review and meta‐analysis of the last 5 years. Oral Dis. 2021;27(8):1881–95.

21. Guan J, Luo Y, Lin Y, Wu Z, Ye J, Xie S, et al. Malignant transformation rate of oral leukoplakia in the past 20 years: A systematic review and meta‐analysis. J Oral Pathol Med. 2023;52(8):691–700.

22. Iocca O, Sollecito TP, Alawi F, Weinstein GS, Newman JG, De Virgilio A, et al. Potentially malignant disorders of the oral cavity and oral dysplasia: A systematic review and meta‐analysis of malignant transformation rate by subtype. Head Neck. 2020;42(3):539–55.

23. Lafuente Ibanez de Mendoza I, Lorenzo Pouso AI, Aguirre Urizar JM, Barba Montero C, Blanco Carrion A, Gandara Vila P, et al. Malignant development of proliferative verrucous/multifocal leukoplakia: a critical systematic review, meta‐analysis and proposal of diagnostic criteria. J Oral Pathol Med. 2022;51(1):30–8.

24. Mohideen K, Ghosh S, Krithika C, Ali-Hassan M, Chole R, Dhungel S. Malignant transformation of proliferative Verrucous Leukoplakia—systematic review & meta-analysis. BMC Oral Health. 2025;25(1):175.

25. de Pauli Paglioni M, Khurram SA, Ruiz BII, Lauby-Secretan B, Normando AG, Ribeiro ACP, et al. Clinical predictors of malignant transformation and recurrence in oral potentially malignant disorders: A systematic review and meta-analysis. Oral Surg Oral Med Oral Pathol Oral Radiol. 2022;134(5):573–87.

26. Palaia G, Bellisario A, Pampena R, Pippi R, Romeo U. Oral proliferative verrucous leukoplakia: progression to malignancy and clinical implications. Systematic review and meta-analysis. Cancers. 2021;13(16):4085.

27. Pimenta‐Barros LA, Ramos‐García P, González‐Moles MÁ, Aguirre‐Urizar JM, Warnakulasuriya S. Malignant transformation of oral leukoplakia: Systematic review and comprehensive meta‐analysis. Oral Dis. 2025;31(1):69–80.

28. Pinto AC, Carames J, Francisco H, Chen A, Azul AM, Marques D. Malignant transformation rate of oral leukoplakia—systematic review. Oral Surg Oral Med Oral Pathol Oral Radiol. 2020;129(6):600-611. e2.

29. Ramos‐García P, González‐Moles MÁ, Mello FW, Bagan JV, Warnakulasuriya S. Malignant transformation of oral proliferative verrucous leukoplakia: A systematic review and meta‐analysis. Oral Dis. 2021;27(8):1896–907.

30. Ramos-Garcia P, Gonzalez-Moles MA, Warnakulasuriya S. Significance of p53 overexpression in the prediction of the malignant transformation risk of oral potentially malignant disorders: A systematic review and meta-analysis. Oral Oncol. 2022;126:105734.

31. Warnakulasuriya S. Oral potentially malignant disorders: A comprehensive review on clinical aspects and management. Oral Oncol. 2020;102:104550.

32. Vergier V, Porporatti AL, Babajko S, Taihi I. Gingival Proliferative Verrucous Leukoplakia and Cancer: A Systematic Review With Meta‐Analysis. Oral Dis. 2025;31(1):50–8.
